# Cellular Prion Protein Conformational Shift after Liquid–Liquid Phase Separation Regulated by a Polymeric Antagonist and Mutations

**DOI:** 10.1021/jacs.4c10590

**Published:** 2024-09-26

**Authors:** Yangyi Liu, Marcus D. Tuttle, Mikhail A. Kostylev, Graham P. Roseman, Kurt W. Zilm, Stephen M. Strittmatter

**Affiliations:** † Department of Chemistry, Yale University, 225 Prospect Street, New Haven, Connecticut 06511, United States; ‡ Departments of Neuroscience and Neurology, Yale School of Medicine, 100 College Street, New Haven, Connecticut 06510, United States

## Abstract

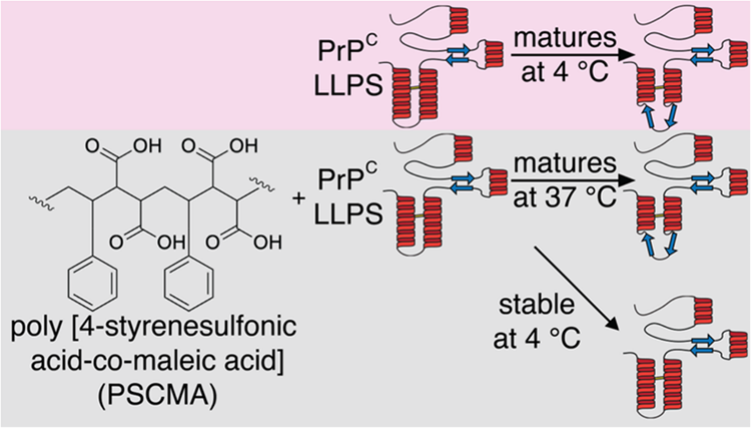

Liquid–liquid phase separation (LLPS) of intrinsically disordered proteins has been associated with neurodegenerative diseases, although direct mechanisms are poorly defined. Here, we report on a maturation process for the cellular prion protein (PrP^C^) that involves a conformational change after LLPS and is regulated by mutations and poly(4-styrenesulfonic acid-*co*-maleic acid) (PSCMA), a molecule that has been reported to rescue Alzheimer’s disease-related cognitive deficits by antagonizing the interaction between PrP^C^ and amyloid-β oligomers (Aβo). We show that PSCMA can induce reentrant LLPS of PrP^C^ and lower the saturation concentration (*C*_sat_) of PrP^C^ by 100-fold. Regardless of the induction method, PrP^C^ molecules subsequently undergo a maturation process to restrict molecular motion in a more solid-like state. The PSCMA-induced LLPS of PrP^C^ stabilizes the intermediate LLPS conformational state detected by NMR, though the final matured β-sheet-rich state of PrP^C^ is indistinguishable between induction conditions. The disease-associated E200 K mutation of PrP^C^ also accelerates maturation. This post-LLPS shift in protein conformation and dynamics is a possible mechanism of LLPS-induced neurodegeneration.

## 1 Introduction

The cellular prion protein (PrP^C^), a cell surface glycoprotein that is highly conserved across mammals, has a central role in neurodegenerative diseases. PrP^C^ can misfold to form the toxic scrapie (PrP^Sc^) conformation causative for infectious prion diseases.^1−4^ PrP^C^ can also interact with other aggregation-prone proteins,^5−8^ such as amyloid-β (Aβ),^8^ and its high affinity for the Aβ oligomer (Aβo) has been known to trigger Alzheimer’s disease (AD) pathophysiology.^9,10^ However, while structural descriptions of the PrP^C^ and PrP^Sc^ forms have been reported, it remains unclear how PrP transitions from one state to another and how its toxicity is achieved.^11−14^

Recent studies on liquid–liquid phase separation (LLPS) have revealed a new facet of macromolecular organization in biology. LLPS facilitates cellular processes ranging from signal transduction^15−19^ to regulation of gene expression^20−24^ and is implicated in neurodegeneration.^25−27^ We showed that PrP^C^ can undergo phase separation under physiological and pathological conditions, but the molecular underpinnings of such LLPS remain unclear.^28^

The LLPS of PrP^C^ is of great interest for further investigation because the interaction between PrP^C^ and Aβo forms a hydrogel phase and can trap metabotropic glutamate receptor 5 (mGluR5), another key player in the AD pathway.^28,29^ Anionic polymers, such as poly [4-styrenesulfonic acid-*co*-maleic acid] (PSCMA), competitively block the interaction between PrP^C^ and Aβo and dissociate the hydrogel phase.^28^ More importantly, PSCMA has been shown to reverse transgenic mice from AD-related memory deficits and block PrP^Sc^ propagation in scN2A cell culture.^30^ These previous findings highlight the biological importance of PrP^C^ phase transitions, as well as the antagonist PSCMA.

In this study, we find that PSCMA can induce a reentrant phase transition of recombinant PrP, similar to the hydrogel formation between Aβo and PrP.^28^ When the concentration of 20 kDa PSCMA is lower than 1/8 that of PrP, PSCMA interacts with PrP only in a condensed liquid phase and leaves the dilute solution-phase PrP unperturbed. Only at a higher PSCMA concentration do the two macromolecular species interact in a homogeneous solution phase. The PSCMA-induced PrP liquid has a morphology similar to that of the previously reported PBS-induced PrP liquid but a lower PrP mobility. Regardless of the presence of PSCMA, PrP liquid undergoes a post-LLPS conformational change that generates more β-sheet content and lowers PrP mobility, a process we describe as “maturation”. However, the presence of PSCMA stabilizes the intermediate state and makes maturation slower. Interestingly, the pathological mutant E200 K has a maturation process much faster than that of WT and any other mutants tested. These findings led us to a deeper understanding of PrP LLPS and suggest a link between PrP phase transitions and neurodegenerative diseases.

## 2 Results

### 2.1 PSCMA Induces Liquid–Liquid Phase Separation of PrP

**Figure 1:**
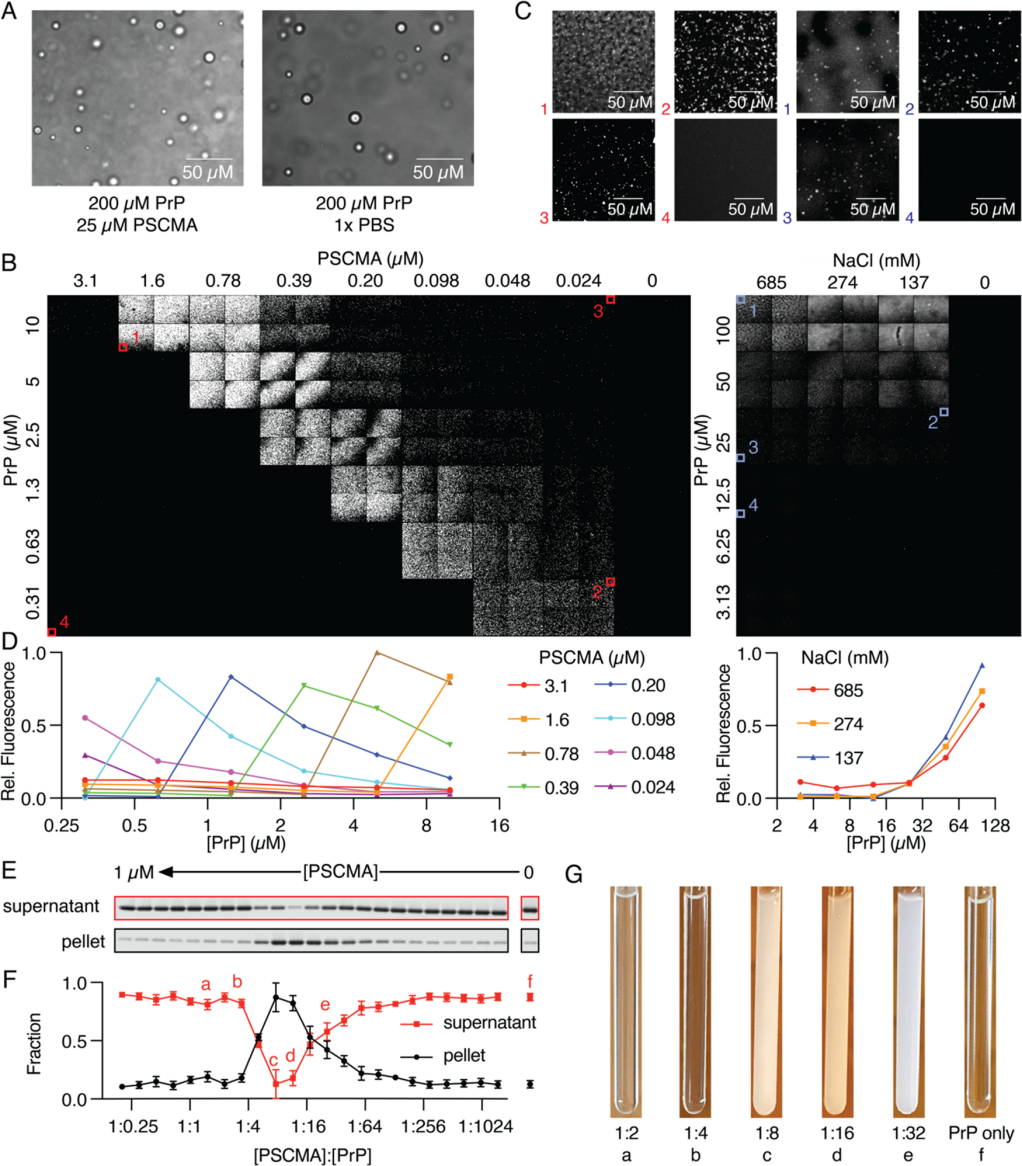
PrP in aqueous solution undergoes phase separation with the addition of PSCMA. (A) Bright-field microscopic images of PrP liquid droplets induced by PSCMA (left) and PBS (right) at 40× magnification. (B) Fluorescence images of PrP LLPS in PSCMA (left) and PBS (right). Each well was supplemented with 50 nM Alexa-488-labeled PrP. PBS-induced LLPS is undetectable when PrP concentration is lower than 25 μM, whereas PSCMA-induced LLPS is still detectable at 0.31 μM PrP. (C) Zoom-in of selected regions in B showing droplets and settled liquid layers. (D) Quantification of total fluorescence intensity of images in B as a function of PrP concentration. (E) SDS-PAGE analysis of supernatant and pellet fractions of a PSCMA–PrP titration. (F) Quantification of the PrP band in supernatant (black) and pellet fractions (red) in (E). Error bars represent SEM, *n* = 3 different titration experiments. (G) Images of PrP with PSCMA at different concentration ratios ([PSCMA]:[PrP]), indicated by lowercase letters a–f on the titration curve in F.

We have shown that PrP undergoes LLPS at concentrations at or above 30 μM in isotonic saline buffered to neutral pH with phosphate, whereas 50 nM PrP undergoes a hydrogel transition in the presence of its Aβo ligand.^28^ The hydrogel transition removes all PrP from the solution at a specific molar ratio to Aβo and eventually dissolves with a sufficient excess of Aβo. Since PSCMA competes with Aβo as a PrP ligand, we assessed its effect on the PrP phase state. When adding PSCMA (average M.W. of 20 kDa) to PrP solutions, we observed that the resulting mixture became turbid, and demixed droplets have a size similar to those of PBS-induced PrP liquid in the absence of other macromolecular species (Figure 1A).

The addition of more PSCMA eventually resolubilizes the mixture, and no droplets are observed. We performed a two-way titration experiment to quantify this reentrant phase separation phenomenon by imaging PrP at different concentrations supplemented with 50 nM Alexa-488-labeled PrP as a fluorescent reporter. At around 1:10 [PSCMA]/[PrP] molar ratio, PSCMA-induced phase transition was detected with as little as 310 nM PrP, whereas PBS-induced liquid can be detected only when protein concentration is ∼100-fold higher, above 25 μM (Figure 1B–D). A confirmatory titration with physical separation was done by mixing 10 μM PrP with different concentrations of PSCMA and analyzing the protein composition of the resulting supernatant and pellet fractions with SDS-PAGE (Figure 1E,F). The optimal [PSCMA]/[PrP] molar ratio that facilitates LLPS was determined to be approximately 1:8, with little LLPS at ratios >1:4 or <1:200. Thus, PSCMA-induced PrP phase transition has a reentrant behavior similar to that of Aβo-induced PrP hydrogel phase transition, but a morphology similar to that of the PBS-induced PrP liquid phase. The absolute PrP concentration dependence is similar to that of Aβo-induced PrP in the submicromolar range.^28^ To further test whether LLPS of PrP is a general consequence expected upon the addition of anionic polymers, the same titration was repeated, substituting 3.4 kDa poly(4-styrenesulfonic acid) (PSS) for PSCMA. The results (Figure S2) are largely the same, with the exception of the extent of LLPS peaks at a [PSS]/[PrP] molar ratio of 1:1.

### 2.2 PSCMA Lowers the Mobility of PrP^C^ in the Condensed Liquid Phase

**Figure 2:**
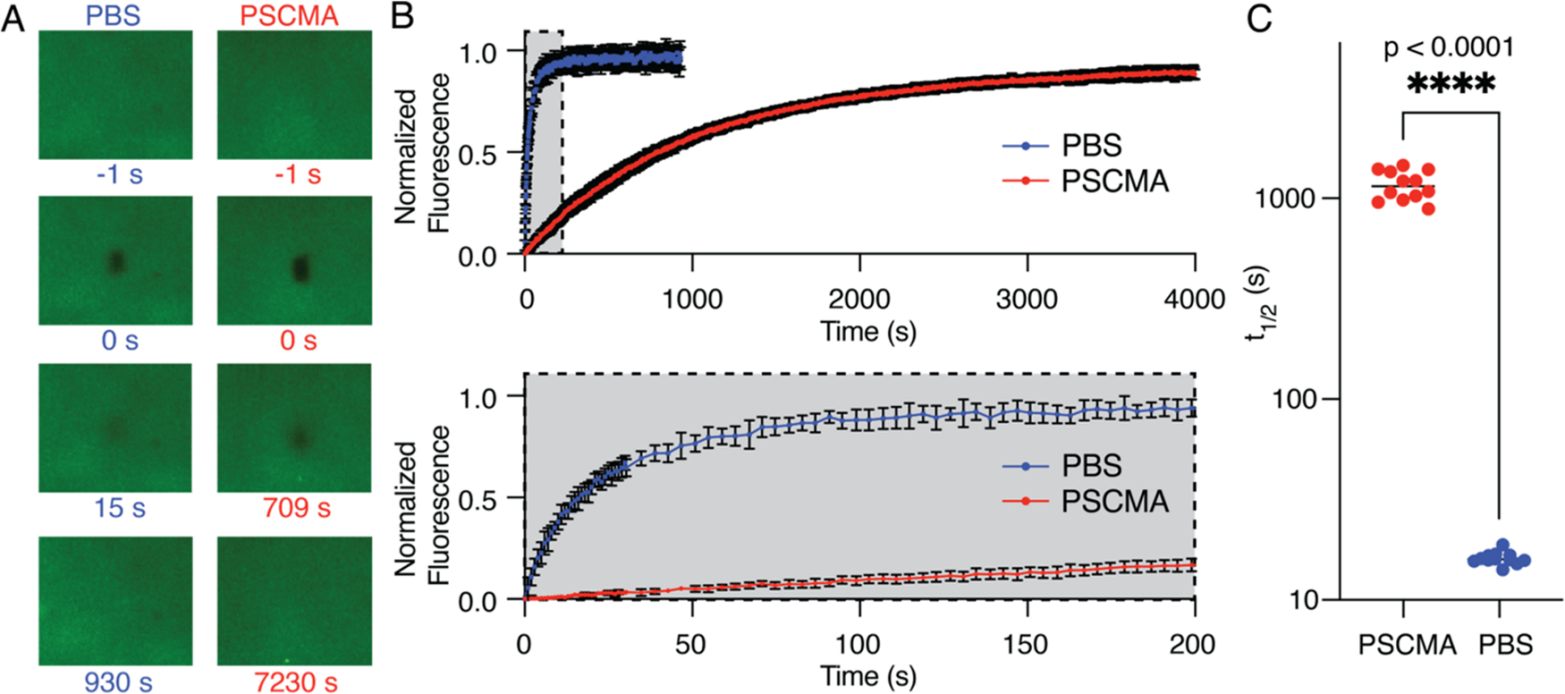
PSCMA lowers the mobility of PrP in the liquid phase, as shown by FRAP. (A) Full recovery was seen in both PBS- and PSCMA-induced liquids. Images show bleach recovery of the 2 μm × 2 μm area. (B) Quantified fluorescence recovery over 4000 s (top) and 200 s (bottom) *n* = 12. Error bars represent SD. (C) Comparison of half-recovery time of PSCMA- and PBS-induced liquids. *n* = 12, *p* < 0.0001.

The viscous liquid nature of PSCMA-induced PrP droplets was confirmed by fluorescence recovery after photobleaching (FRAP). Induced at a 1:8 molar ratio, PrP liquid supplemented with the Alexa-488-tagged protein was allowed to settle for 1 h to the bottom of the chambered glass and full recovery was seen after photobleaching, similar to the previously described PBS-induced PrP liquid (Figure 2A). However, a large reduction in mobility was seen with an almost 100-fold difference in the half-recovery time, highlighting the difference between the two liquid states (Figure 2B,C).

### 2.3 PrP in Complex with PSCMA Can Only Be Observed at PSCMA Concentrations Higher than the 1:8 Molar Ratio

**Figure 3:**
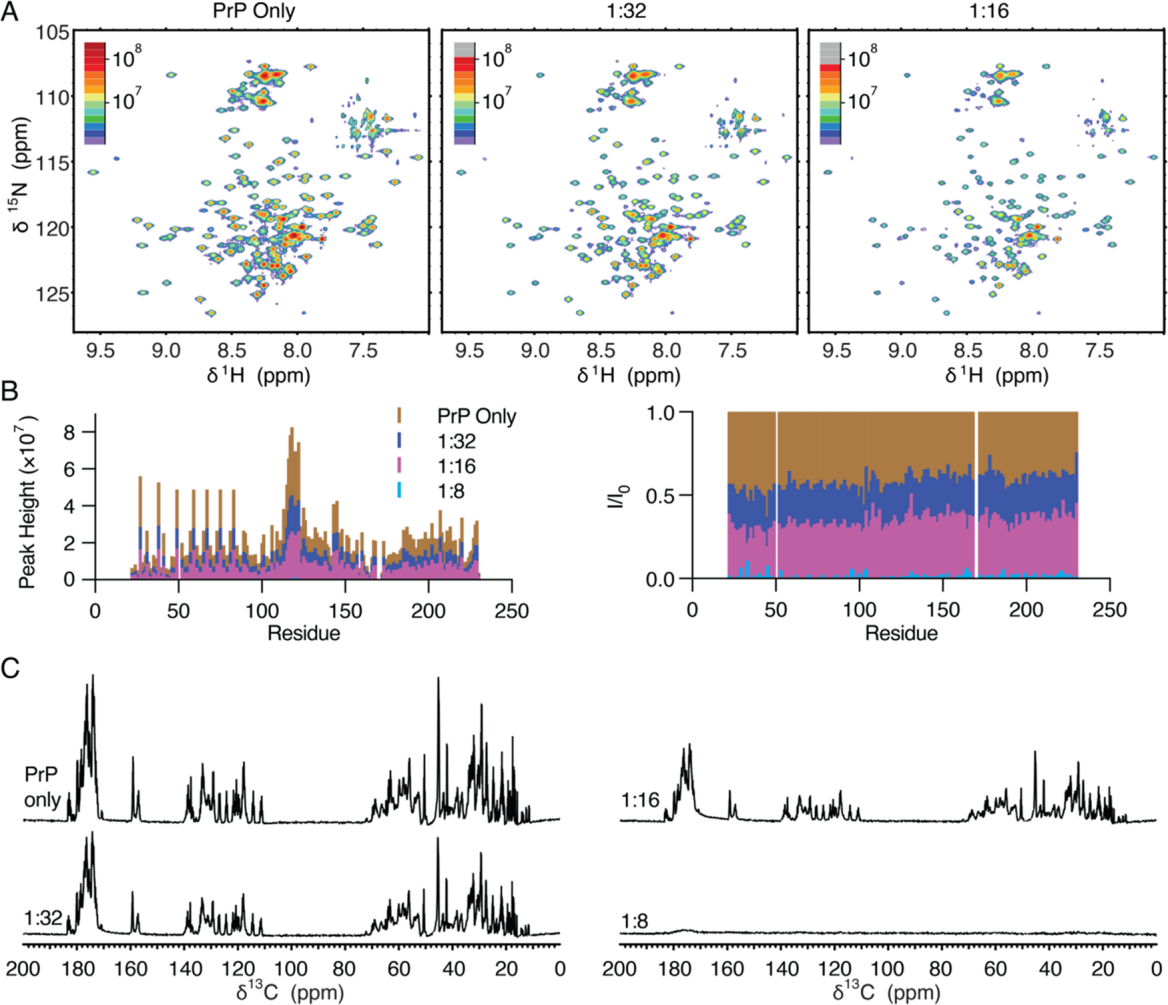
PSCMA does not alter the conformation of PrP at concentration ratios of 1:32 and 1:16 in the dilute solution phase. (A) ^1^H–^15^N HSQC solution NMR spectra on PrP only, 1:32 and 1:16 samples under the same experimental time and conditions. Chemical shift perturbations were not observed in the 1:32 and 1:16 spectra. The color scale shows the absolute signal intensity. No significant signal was observed in the 1:8 sample, and the spectrum is therefore not shown. (B) The intensity plot of each residue (degeneracy-adjusted) on HSQC spectra (left) and the intensity ratio of each residue to its *I*_0_ in the PrP-only spectrum (right). Peak intensities decreased uniformly across all residues. Gaps in both plots come from proline or unassigned residues. (C) ^13^C one-pulse solution spectra of PrP^C^ only, 1:32, 1:16, and 1:8 under the same experimental time, indicating the decrease in the concentration of unbound PrP in solution.

Multiple uniformly ^13^C, ^15^N-labeled PrP samples were prepared to study its conformation upon interaction with PSCMA at different molar ratios (Figure 1G). Solution ^15^N–^1^H HSQC experiments on 1:32 and 1:16 samples yielded the same spectra as the PrP-only (apo) preparation but with significantly reduced intensities, while the 1:8 sample with maximal LLPS did not produce a spectrum with a reasonable signal-to-noise ratio under the same acquisition time (Figure 3A). These spectra indicate that the condensed liquid phase produces little or no signal under these conditions, consistent with the PrP being sequestered in a phase too viscous for solution-phase NMR. Presumably, very little PSCMA remains in the fluid phase, and therefore, the NMR spectra detect only unperturbed PrP that has not partitioned into the liquid droplets. Using an NMR pH titration to extrapolate to our sample conditions, we were able to utilize chemical shift assignments previously published by Zahn et al.^11^ On this basis, we identified all resolved peaks in our spectra and could quantify the intensity of each residue. The quantification shows a uniform reduction in the signal across all residues (∼40% reduction from apo to 1:32, ∼70% to 1:16, and ∼100% to 1:8), indicating that only the unbound form of PrP^C^ is present in the dilute solution phase, while PrP bound to PSCMA has separately condensed into the liquid droplets before the 1:8 molar ratio (Figure 3B). ^13^C 1D spectra independently confirm this result in PrP^C^ only, 1:32, 1:16, and 1:8 samples (Figure 3C).

### 2.4 N-Terminal Lysine Residues are Responsible for PSCMA–PrP Binding

**Figure 4:**
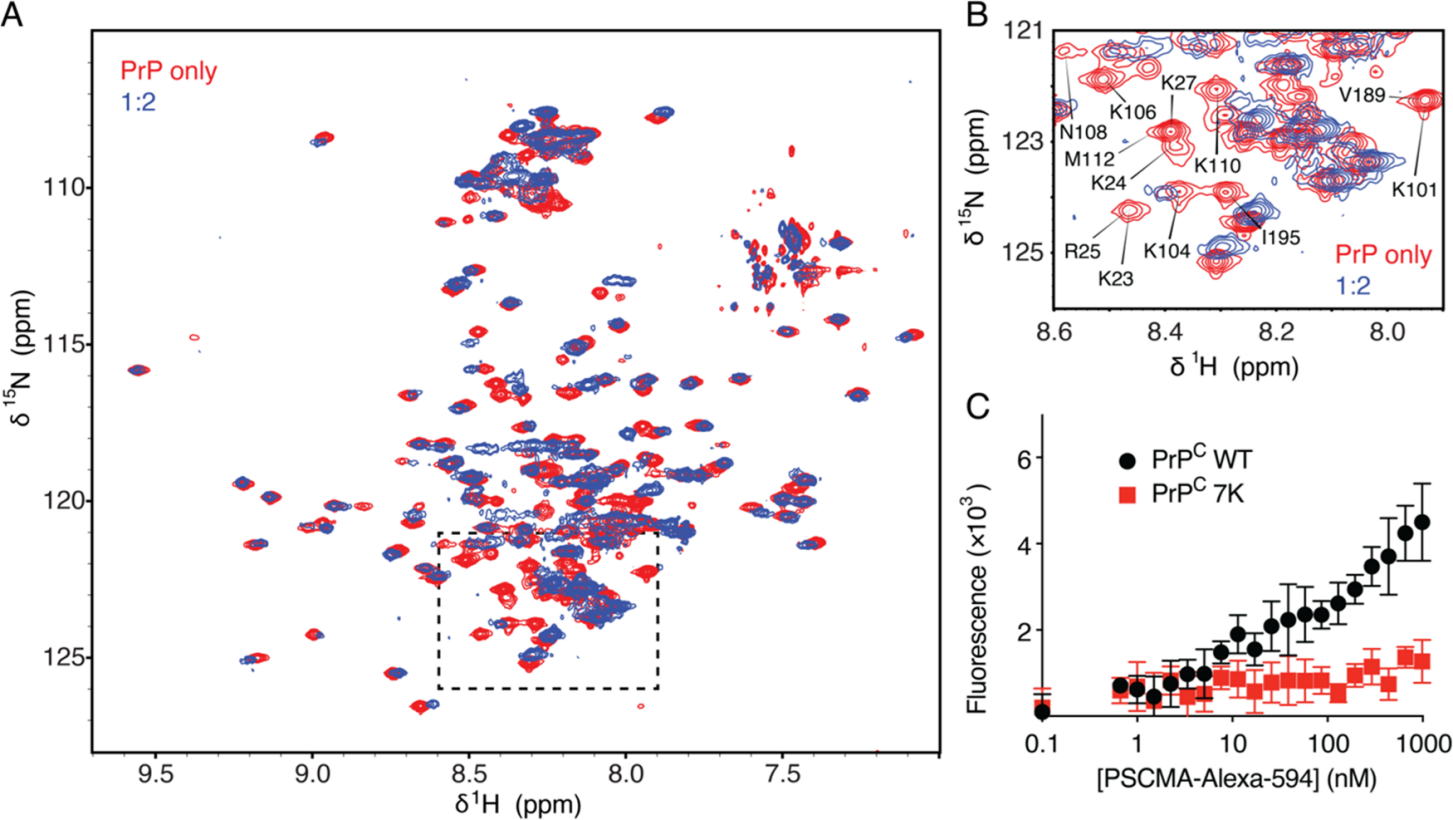
Two lysine patches in the N-terminal domain are important for PSCMA–PrP interaction. (A) Overlay of PrP^C^ only (red) and 1:2 (blue) ^15^N–^1^H HSQC spectra with the same experimental time and conditions under 800 MHz. (B) Zoom-in of the dotted lysine region: 6 out of 7 N-terminal lysine residues are undetectable upon PSCMA addition. (C) Plate-based binding experiment of PrP to Alexa594-labeled PSCMA. PrP 7K mutant (K23, 24, 27, 101, 104 106, 110A) abolishes binding to PSCMA *n* = 6. Error bars represent SD.

PSCMA solubilizes PrP to a pure solution phase at 1:4 and 1:2 molar ratios (Figure 1E–G). This single phase at a high PSCMA concentration has a viscosity in between that of the NMR buffer and the LLPS droplets and is fluid enough to permit interrogation by solution-phase NMR methods, albeit at some intensity loss (∼30%) relative to the apo sample. From the overlay of ^15^N–^1^H HSQC spectra of PrP^C^ only and 1:2 samples, the conformation can be judged to be very similar in much of the protein, with rather small shifts observed for the well-resolved peaks in the presence of PSCMA. Binding in the intermediate exchange regime is indicated by the disappearance of several peaks upon PSCMA addition (Figure 4A). Based on our extrapolation of the published assignments from Zahn et al.,^11^ we identified these missing signals and found that 6 out of the 7 lysines in the N-terminal domain are undetectable in the presence of PSCMA, suggesting that they are involved in PSCMA binding (Figure 4B). This is consistent across samples with different pH values (Figures S3 and S4). To confirm this result, we performed a plate-based binding assay with fluorescently labeled PSCMA on both WT PrP and a mutant that silences all 7 N-terminal lysine residues to alanine (7K). These data (Figure 4C) confirm that these lysine residues are essential for binding between PSCMA and PrP.

### 2.5 PSCMA-Induced PrP Liquid has an Irreversible Temperature-Dependent Maturation Process

**Figure 5:**
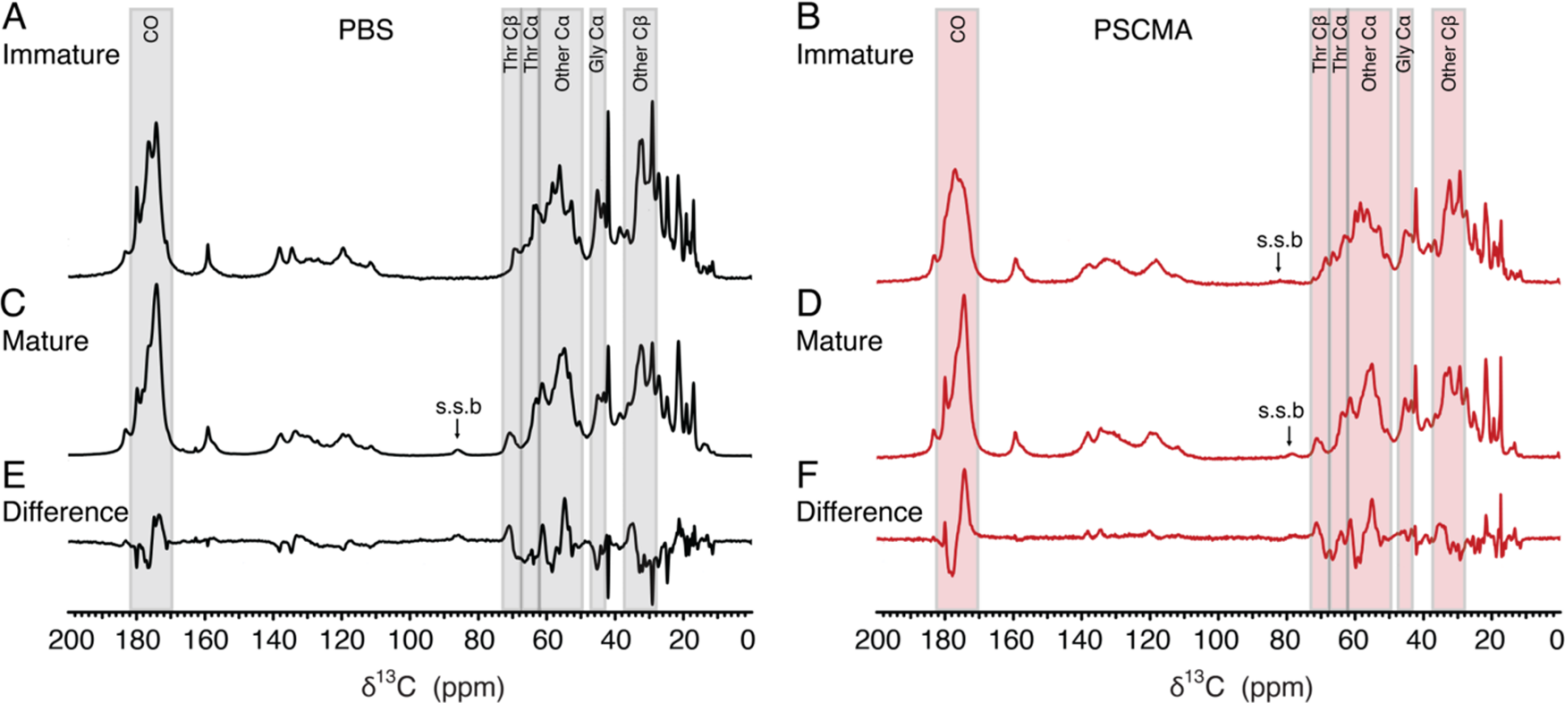
Both PBS- and PSCMA-induced PrP liquids undergo maturation and have a conformational change. ^13^C direct-polarized 1D MAS ssNMR spectra were taken at 800 MHz. (A) PBS-induced PrP liquid at 4 °C in its initial immature phase. (B) PSCMA-induced PrP liquid at 4 °C in its initial immature phase. (C) PBS-induced PrP liquid at 4 °C in its matured phase. Maturation occurred spontaneously at 4 °C over 48 h. (D) PSCMA-induced liquid at 37 °C in its matured phase. Maturation occurred only with temperature increase. (E) Spectral difference of matured and immature PBS-induced PrP liquid highlighting structural changes in threonine, carbonyl carbon, and part of α- and β-carbon regions. (F) Spectral difference of matured and immature PSCMA-induced PrP liquid highlighting similar structural changes.

We observed that the condensed PrP liquid phase (centrifuged to exclude the dilute solution phase) induced by PSCMA is stable at 4 °C for at least 1 week (Figure S5A). However, the protein conformation changed substantially when the temperature was raised from 4 to 37 °C (Figure 5B,D). Lowering the temperature back to 4 °C did not reverse this maturation process (Figure S5B). By taking the difference between the immature and mature sample spectra, we are able to identify more clearly the conformational changes that occur during maturation. Backbone CO and Cα ^13^C chemical shifts are primarily determined by torsion angles and therefore reflect where a particular residue falls in Ramachandran space. Quite generally, the ^13^C CO chemical shift for a helical residue is 1–2 ppm downfield (higher ppm) from the β sheet or a random coil conformer. The chemical shift changes for Cα and Cβ centers upon loss of helicity are about double this, with Cα shifting downfield 2–4 ppm while Cβ carbons shifting upfield a similar amount.^31^ The ^13^C spectrum of a protein then, in large part, is a reflection of local elements of the secondary structure. When a helix becomes a random coil, the CO band is expected to shift its center of gravity 1–2 ppm lower on average. This in fact is just what is observed in the carbonyl region of difference spectra (mature–immature) in Figure 5E,F, an overall loss in helical character with a concomitant increase in residues adopting either random coil or β-sheet conformations. During maturation, a large fraction of the threonine residues also switch from α-helical to random coil or β-sheet as shown in the peak shifts of threonine Cα and Cβ carbons between 65 and 73 ppm. Other Cα species (excluding Thr and Gly) between 50 and 65 ppm are observed to have large conformational changes given the similar dispersive line pattern observed in the difference spectra, although the exact amino acid identities are not easily discernible due to chemical shift overlap in this region.

### 2.6 PBS-Induced PrP Liquid has a Similar Maturation Process but with a Less Stable Intermediate State

**Figure 6:**
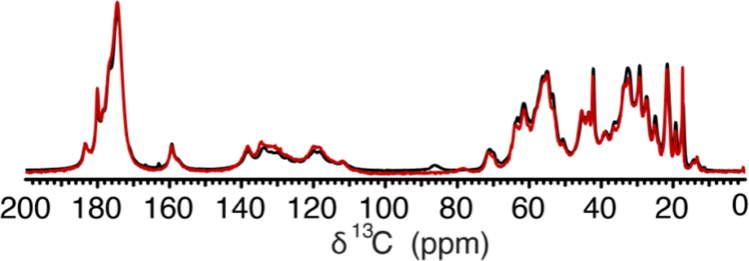
Two matured PrP liquids have the same conformation regardless of the induction method. Figure shows an overlay of ^13^C 1D ssNMR spectra of matured PBS-induced (black) and PSCMA-induced (red) PrP liquid.

PrP liquid induced by PBS matured spontaneously over 48 h at 4 °C (Figure 5A,C), showing much less stability than PSCMA-induced PrP liquid. This maturation process involves conformational changes similar to those of the PSCMA-induced liquid (i.e., threonine, Cα, and carbonyl regions, Figure 5E). The two matured liquids have virtually the same conformation regardless of the presence of PSCMA (Figures 6 and S6).

### 2.7 PSCMA-Induced E200 K LLPS Is More Prone to Maturation

**Figure 7:**
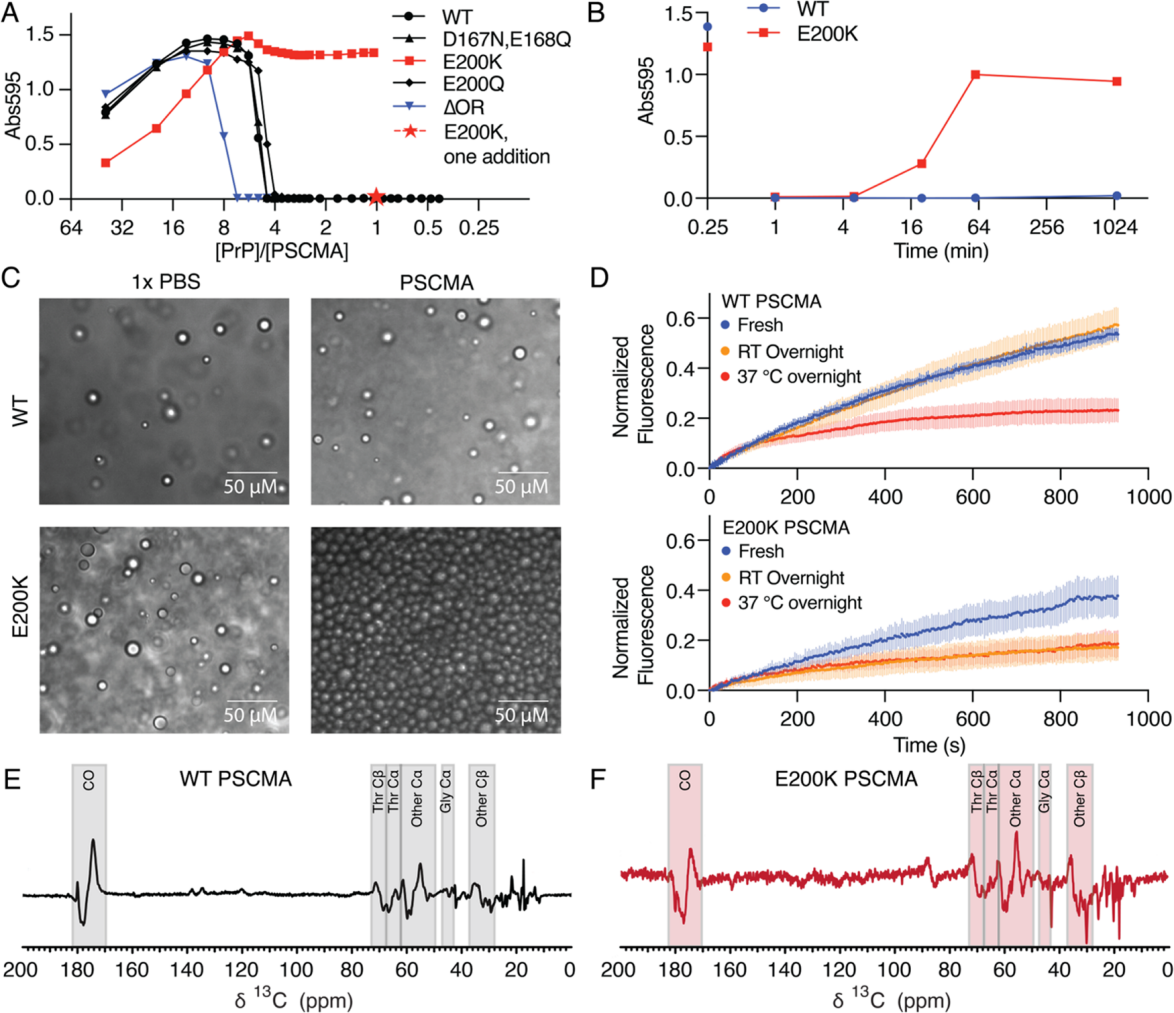
PSCMA-induced LLPS of pathological mutant E200 K has a short-lived intermediate state and is more prone to maturation. (A) The turbidity assay on mutants in sequentially added PSCMA. E200 K cannot be rehomogenized in a high concentration of PSCMA if sequentially added but stays as a clear solution if PSCMA is added only once to achieve the same concentration. (B) Kinetic experiment showing the irreversibility of E200 K LLPS by a second dose of PSCMA after an incubation period longer than 20 min. (C) PSCMA-induced E200 K liquid has a dense population of droplets, different from other samples. (D) Mobility of PSCMA-induced WT PrP liquid does not change unless the temperature is increased (top), but E200 K liquid becomes rigid spontaneously under room temperature incubation (bottom). Error bars represent SD, *n* = 9. (E) Spectral difference of matured PSCMA-induced WT liquid at 37 °C minus the immature state at 4 °C. (F) 3× spectral difference of PSCMA-induced E200 K liquid at 39 h minus the initial state at 2 h, showing conformational changes similar to WT. This process happens spontaneously at 4 °C. The dispersive feature at ∼90 ppm are sidebands, a consequence of the spectra being subtracted having been acquired at slightly different spin rates.

We tested several PrP mutants to further investigate the reentrant LLPS behavior induced by PSCMA. Our mutant library includes a pathological mutant E200 K and its biochemical control E200Q, octarepeat deletion (ΔOR), and a double mutation D167N-E168Q that silences the negative-charge patch between helices I and II. The turbidity assay, which is a common method used for studying protein LLPS,^32,33^ was performed on each mutant in the presence of PSCMA. Specifically, PSCMA was sequentially added to the same protein sample to achieve different concentration ratios, and the turbidity reading was taken after each dose of PSCMA addition. Most mutants tested showed reentrant LLPS behaviors similar to those of WT, with ΔOR showing a shifted critical concentration ratio (Figure 7A). The only exception is the pathological mutant E200 K, which remained turbid in a heterogeneous state even at a molar ratio of 1:1 (Figure 7A, red squares). However, directly adding one bolus of the same total amount of PSCMA to a fresh E200 K sample resulted in a clear solution (Figure 7A, red star). This led to a hypothesis that the E200 K liquid has a very short-lived intermediate state that cannot survive the duration of the experiment (about 30 min) where PSCMA was sequentially added.

We further investigated the existence of an intermediate state of E200 K with a simple kinetic experiment. At time zero, five LLPS samples were prepared by adding PSCMA to achieve a 1:8 ratio, followed by an incubation period. After incubation for 1, 5, 20, 60, or 1080 min, the second dose of PSCMA was added to each LLPS sample, respectively, to achieve a 1:1 ratio to test the reversal of the LLPS back to a clear solution. WT PrP liquid can be recovered back to solution by PSCMA after any incubation time tested (Figure 7B, blue). However, E200 K LLPS can only be partially recovered after 20 min and cannot be reversed at all after 60 min (Figure 7B, red). Under a bright-field microscope, PSCMA-induced E200 K LLPS also stands out for its dense population of unmerged droplets, even immediately after the induction (Figure 7C, lower-right). FRAP further shows that PSCMA-induced E200 K liquid becomes rigid more easily, simply by incubation at room temperature (Figure 7D, bottom), whereas the WT liquid preserves its fluidity under the same conditions and becomes rigid only after 37 °C incubation (Figure 7D, top). As detected by ssNMR, conformational changes associated with this maturation process of PSCMA-E200 K liquid are similar to that of WT (Figure 7E,F). However, PSCMA-E200 K liquid matured spontaneously at 4 °C, whereas PSCMA-WT liquid matured only when the temperature was raised to 37 °C, further highlighting the instability of the PSCMA-E200 K intermediate liquid state.

## 3 Discussion

This study delves into the complexity of PrP LLPS and creates a link between PrP LLPS and PrP^C^ pathology. A major finding is that PrP continues to have conformational changes after LLPS, and the rate of this process is dependent on the temperature, presence of PSCMA, and mutation.

### 3.1 PSCMA is a Potent PrP LLPS Inducer that Competes against Aβo

The polymer PSCMA was initially discovered to target the interaction between PrP^C^ and Aβo and has been shown to dissociate the PrP^C^–Aβo hydrogel phase.^28,30^ In this study, we found that PSCMA can also induce the reentrant phase transition of PrP and lower the saturation concentration *C*_sat_ to the submicromolar range. LLPS of PrP is initiated when PSCMA is present in small quantities and plateaus around 1:8 molar ratio (PSCMA/PrP). With more PSCMA, the suspension is reversed to a homogeneous solution.

**Figure 8:**
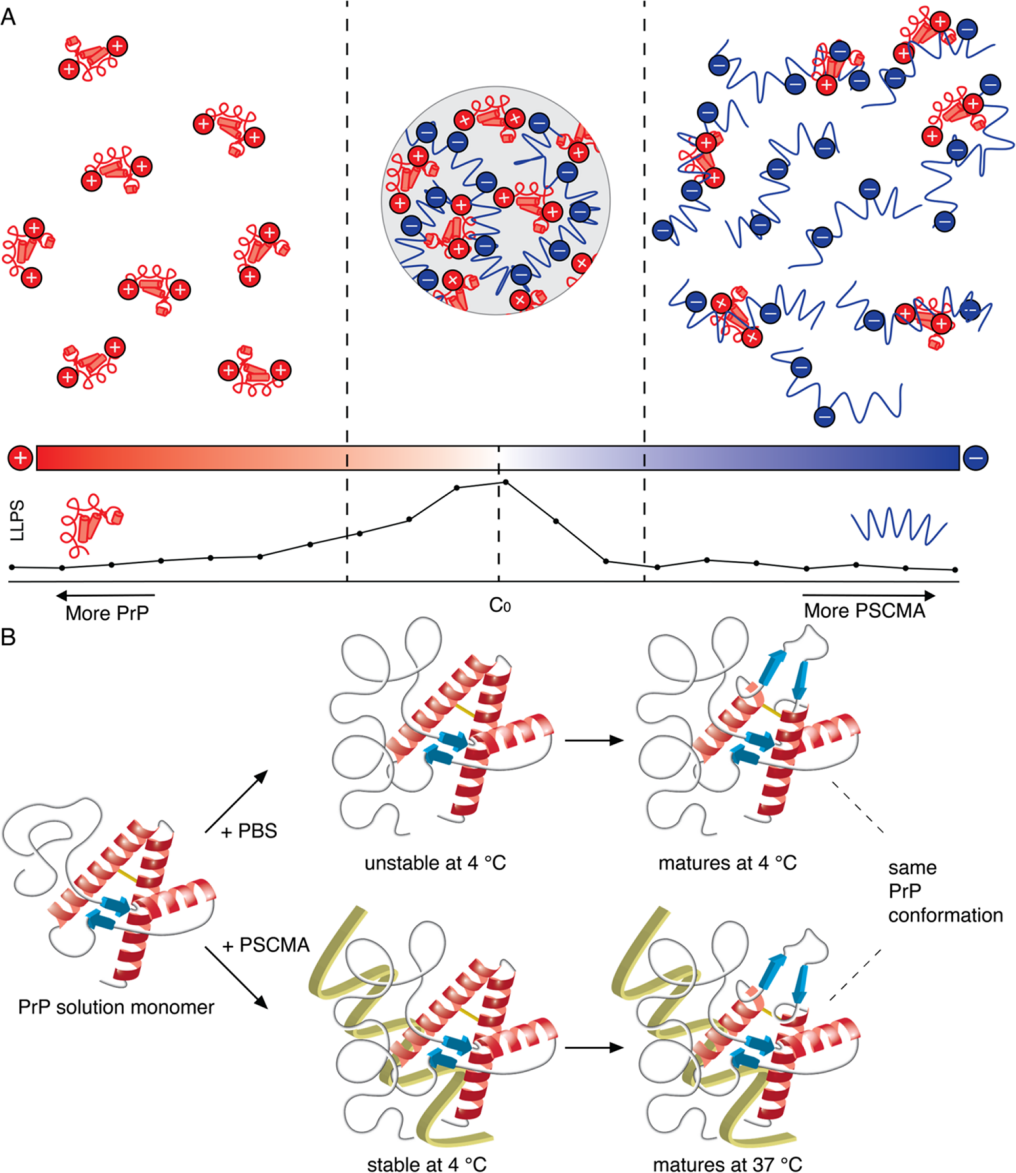
Schematic summary of the effects of PSCMA on PrP LLPS. (A) PSCMA (blue) induces reentrant LLPS of PrP (red) through a charge inversion mechanism. (B) PSCMA (yellow) delays the maturation of phase-separated PrP by stabilizing an intermediate state. PrP matures to the same partial β-sheet conformation regardless of the induction method.

This reentrant mode of phase transition has been described in other LLPS systems, such as FUS and Tau regulated by RNA,^34−38^ TDP-43 by small molecules,^39,40^ and PrP by Tau and α-synuclein.^6,7^ Previous research has attributed this reentrant LLPS behavior to multivalent electrostatic interactions between molecular species, such as polycation-controlled reentrant LLPS of acidic proteins^41−43^ and triphosphate or RNA (polyanion)-controlled reentrant LLPS of alkaline proteins.^34,36,44,45^ In this model, the multivalent electrostatic attraction is responsible for the initial phase separation at low ligand concentrations, which peaks at a concentration of *C*_0_ where charge inversion occurs. At higher concentrations, the phase-separated coacervate dissolves due to charge screening by excess anionic polymer additive. No LLPS is observed at high concentrations as the anionic polymer molecules interact with only a single cationic protein at a time, and electrostatic repulsion prevents phase separation.^36,46^ Our reentrant LLPS system of PrP (cationic) and PSCMA (anionic) is consistent with this basic model (Figure 8A). The NMR and binding assay results in this work and our previous study of Aβo binding by PrP^28^ identify the lysine clusters at 23–27 and 101–110 as the primary cationic sites in PrP responsible for binding to PSCMA (although additional interactions are not ruled out).

Some insight into the scaling of the principal ionic molecular interactions that drive this reentrant phase transition is provided by comparison to the results obtained using 3.4 kDa PSS. Both PSCMA and PSS are anionic polymers with negatively charged side chains arranged along a linear saturated hydrocarbon backbone (Figure S2A). When prepared at neutral pH, both polymers have the same number of charged sites per unit length decorated by condensed charge compensating cations (Na^+^). In the case of 3.4 kDa PSS, the *C*_0_ with PrP occurs at [PSS]/[PrP] ∼ 1:1 (Figure S2). At this molecular weight, the PSS has, on average, 16 monomers and a backbone of about 32 carbons in length. One possible molecular arrangement at *C*_0_ in the coacervate phase would be PrP acting as a bidentate cation, with the two positively charged lysine clusters in the N-terminal domain bridging between different PSS chains to clusters of negative charge. If the 1:1 ratio is a result of this chain length being the minimum required for either steric or charge repulsion forces, the ratio of [polymer]/[PrP] on a molar basis is expected to decrease as the chain length of the polymer length increases. The PSCMA used in this study at 20 kDa has the order of 58 monomers and an average backbone of 232 carbon centers, or 7.25 times as long as the PSS. If the same sort of molecular model applies with 20 kDa PSCMA as with 3.4 kDa PSS, the *C*_0_ [PSCMA]/[PrP] ratio would be predicted to be ∼1:7.25, consistent with the experimentally observed value of ∼1:8. One possible picture is that on a single PSCMA chain, about 8 PrP molecules can have one of the charged lysine clusters displace Na^+^ as the condensing cations, spaced on average 7 or 8 monomers apart along the length of the chain. The other PrP lysine cluster then binds to a different PSCMA molecule, leading to the formation of a maximally entangled polymer network. In either system, when the polymer concentration is increased to the point that there are not enough PrP molecules to support this interpenetrating entangled network, the coacervate dissolves. The excess PSCMA molecules then interact with a single PrP at a time, and the phase is rendered fluid by the dominant electrostatic repulsion of the PSCMA. This behavior is also consistent with our prior observations on hydrogel formation between PrP and Aβo. In that instance, the Aβo is the polyvalent anionic component, which appears to support PrP binding to as many as 4 negatively charged patches on their roughly spherical surfaces. At C_0_, the PrP bridges between pairs of oligomers, leading to the observed stoichiometry of 1:2 (Aβ monomer/PrP).^27^

### 3.2 PSCMA Impedes β-Sheet Formation of Phase-Separated PrP

We found that both PSCMA-induced and PBS-induced PrP liquids undergo conformational changes after LLPS (Figure 8B). In both cases, the protein conformation appears to lose α-helical character as it matures. By this, we mean that there is a reduction in the number of residues having helical torsion angles. Our NMR data do not distinguish between nascent helices, helical turns, or fully formed α-helices. In the absence of site-specific assignments, the ^13^C shifts only provide insight into which of the canonical sets of torsion angles a residue has adopted. Overall, it is very clear that a significant number of residues with helical torsion angles have changed conformation. In the PSCMA LLPS, this shift involves ∼12% of the 210 aa in PrP, or about 25 residues, as judged from intensities in the difference spectra. Of these, about 8 are threonines, identified by their unique downfield Cβ chemical shift and their share of the intensity in the spectral difference. This represents 2/3 of the 12 total threonines in PrP. Since a string of 8 helical threonines are located closely together in the stretch from aa 183 to 201 at the end of helix II and the beginning of helix III, this likely reflects the unfolding of the juncture of these two helices. Another 8 intervening 8 helical residues interspersed within these threonines would also follow this conformational shift for a total of 16 aa. Where the remaining 9 residues are located is more speculative. It is interesting to note that there are only 35 amino acids in the helix-turn-helix run connecting the disulfide bond between cysteines 179 and 214. The unwinding of this connecting helical segment is one possible way to facilitate β-sheet formation without breaking the disulfide bridge.

Beyond accounting for the number of residues involved in the loss of helical character, it is difficult to differentiate whether this is specifically a gain in random coil or β-sheet content since the ^13^C chemical shifts for these are less distinctive in the absence of sequential assignments. MAS ^13^C–^13^C correlation spectra of the matured states (Figure S6) indicate that the alanines, largely localized in the palindrome, have clearly adopted random coil conformations. The Cα-CO glycine cross peak is another easily identified outlier in these spectra and shows no indication of any helical content. Unfortunately, glycine shifts for the random coil and β-sheet are quite similar, so these data cannot confirm β-sheet formation but only the absence of helical character.

Several other observations, however, indicate a significant reduction in molecular mobility during maturation, and this is most readily explained by the formation of nascent β-sheets, thereby making the coacervate phase much more rigid. The first observation is the sizable reduction in fluorescence recovery in FRAP (Figure 7D) upon incubation at 37 °C, indicating that some portions of the sample now have a viscosity consistent with a molecular solid. In addition, the immature coacervate is observed by NMR to initially have large amplitude motions, which provide for significant motional narrowing of the anisotropy in the ^13^CO chemical shift. Because of this mobility, the ^13^C MAS spectra of the immature samples do not have the characteristically intense spinning sidebands expected for rigid carbonyls. Upon maturation, this mobility is reduced, and the spinning sideband intensity increases, indicating higher sample rigidity (Figure 5). Lastly, the presence of the β-sheet content in matured samples was confirmed by observing a significant thioflavin T (ThT) fluorescence signal (Figure S7). Since the intensity of ThT fluorescence is affected by both the kinetics of phase separation and drop coalescence and settling and our plate reader assays do not support low-temperature operation, it did not prove practical to follow the maturation kinetics using ThT fluorescence on samples comparable to the centrifugally separated samples studied by NMR. Nevertheless, both PBS and PSCMA LLPS immature samples are found to reproducibly produce the same ThT fluorescence signal when fully matured. Taken together, these observations are consistent with significant β-sheet formation in the final stage of maturation.

The presence of PSCMA does, however, appear to greatly impede β-sheet formation by either making folding intermediates or the initial PrP^C^ condensed phase much more stable, prolonging its lifetime from 2 days to greater than 7 days at 4 °C (Figure S5A). The maturation in the presence of PSCMA was observed only at 37 °C, presumably due to greater dynamics at higher temperatures facilitating PrP^C^ rearrangement. In our initial report on the discovery of PBS-induced LLPS of PrP, we were unaware of this rapid maturation phenomenon.^28^ Only when we observed maturation in the presence of PSCMA did we recognize this as a possibility. With this knowledge, we modified the centrifugal concentration and sample transfer procedures to make them as fast and cold as possible, and even with our best efforts, the sample used for the spectrum in Figure 5A is unavoidably partially matured. Although PBS LLPS is fully reversible prior to maturation, as evidenced by faster optical measurements,^28^ longer experiment times and higher temperature incubation will lead to the observation of the onset of β-sheet formation and growth of insolubility.

A particularly striking result in our present study is that PrP in the matured state is largely in the same conformation, regardless of the presence of PSCMA. A size-exclusion chromatography analysis was performed to verify that PSCMA still colocalizes with PrP in the condensed phase upon maturation and does not migrate to the dilute solution phase or otherwise become phase-segregated (Figure S8). Given the similarity of the end-state conformations, it is interesting to compare the immature states more closely to the PrP monomer in solution prior to LLPS induction. Most informative are the differences in the carbonyl region of the ^13^C NMR spectra (Figure S9). Above 180 ppm are resonances for side chain carbonyls, while the backbone carbonyls are largely contained in the two bands centered at 176.7 and 174.3 ppm that align with carbonyls having helical and nonhelical torsion angles, respectively. The loss of helical character is evident from the intensity of these two bands on the progression from immature to mature LLPS states. The intensity ratios of these two bands are also suggestive that the PSCMA-stabilized LLPS state has perceptibly more helical character than the monomeric state in solution (Figure S9).

Given the rapidity of the maturation in PBS-induced liquid, one can easily infer that the PBS-induced liquid also likely started out with as much, if not more, helical character as the monomeric solution. Together, these observations imply that the species that phase separates could be a PrP conformation that is slightly enriched in helical content relative to the native monomeric state. More insights into the principle could be gleaned from MAS ^13^C–^13^C correlation spectra; however, we have not been successful in observing strong cross-peaks for immature samples. Nevertheless, in the dense coacervate, it is likely that the N-terminus is more restricted in mobility in the absence of complete solvation and will find itself needing to associate in some manner with the folded C-terminal domain. Such an association and adoption of structure has been inferred by Zahn for monomeric PrP at the elevated pH used in these LLPS samples.^47^ In that work, pH-dependent NOEs detected the association of the N-terminus with the globular domain and identified a novel folded state of the octarepeat (OR) region in a more compact form of the monomer in solution.^47^ The large reduction in random coil character will result in marginally more aa adopting helical torsion angles consistent with our immature PSCMA spectra (Figures 5B and S5A). Footprinting data also indicate the presence of an equilibrium conformer with preformed metal binding sites, likely the same state detected here by NMR.^48^ Taken together, this suggests that PSCMA-induced phase separation occurs initially by PSCMA ligating and collecting the compact PrP conformer. Once sequestered in a more concentrated coacervate, the PrP can change conformation, driven by more favorable interactions between PrP molecules. In PBS, the same compact PrP conformer appears to be favored by higher pH, and aggregation of the PrP has been suggested to then be a result of interactions between the accompanying structured OR domains. Once LLPS occurs and concentrates the compact PrP conformer, the same maturation process is facilitated. Both coacervates then can mature to the same final matured state that we have identified as β-sheet-rich on the basis of the reduced mobility, insolubility, and ThT fluorescence.

While PSCMA facilitates LLPS, at the same time, it stabilizes the sequestered compact PrP folding intermediate and slows the maturation kinetics (Figure S5). This stabilizing property is reminiscent of a role that has been proposed for polyphosphate, another protein reentrant LLPS inducer that has been reported to act as a chaperone for protein unfolding intermediates and prevent them from further aggregation.^49,50^ Here, we observe maturation to a β-sheet-rich enriched form even in the presence of PSCMA. This suggests that continued interaction with PSCMA during the maturation process may provide a mechanism to steer this eventual aggregation toward nontoxic isoforms that could be otherwise accessible.

The presence of a compact PrP conformer, perhaps in equilibrium with the canonical extended N-terminal PrP, as a common folding intermediate is an attractive unifying concept consistent with a wide range of experimental observations. The variety of ways to induce LLPS can be collectively understood as conditions that shift this equilibrium to phase separate the compact form, which further requires the equilibrium to shift in the remaining supernatant phase.

### 3.3 Fast Maturing Mutant E200 K and the Biological Implications of PrP^C^ LLPS

Disease-related mutants of most LLPS proteins showed reduced mobility compared to their respective WTs and are more prone to liquid-to-solid transitions, such as Tau, FUS, and UBQLN2.^51−53^ In this study, we showed similar results for E200 K, a pathological mutant of PrP^C^. With the stabilizing effect of PSCMA, we were able to capture the intermediate state of E200 K LLPS with a lifetime of roughly 1 h determined by the kinetic turbidity experiment, which is much shorter than that of WT PrP. Compared to WT, the ssNMR spectral changes for E200 K maturation are less prominent due to the failure to capture the majority of immature population after the initial 2 h required to pack the phase-separated sample into the rotor. As exhibited by the first spectrum of the E200 K sample, the conformation already resembles the WT matured state (Figure S10A). It is possible that the charge reversal mutation weakens the negative surface charge of the C-terminal domain, lowering the energy barrier for PrP intermolecular association, thus promoting the rate of β-sheet formation. The instability of the intermediate state of pathological mutant E200 K, coupled with the fact that the antagonist PSCMA stabilizes the PrP intermediate state, leads to a hypothesis that the PrP intermediate state we observed might be an entry point to a pathway where it can still be rescued by PSCMA or other reentrant LLPS inducers before further misfolding or aggregation.

## 4 Conclusions

PrP has a post-LLPS maturation process that involves lower protein mobility and a conformational change. PSCMA, an antagonist of PrP-Aβo, can induce reentrant LLPS of PrP that also undergoes the same maturation process, but PSCMA stabilizes the initial protein conformation in the condensed phase, delaying maturation. Taken together, the observation on LLPS and maturation kinetics suggest the equilibrium presence of a compact minor conformer of PrP that is preferentially sequestered in phase separation mediated by either PBS or PSCMA. The pathological mutant E200 K is predisposed to mature in the phase-separated state, undergoing a conformational change similar to WT, and matures very quickly, even in the presence of PSCMA. These findings provide new insights into the molecular mechanism of PrP^C^ LLPS and its biological significance.

## Supporting Information Available

Experimental Methods; SDS/PAGE; and ^15^N–^1^H HSQC NMR, ^13^C NMR, SEC, and ThT data (PDF)

## Abbreviations

LLPS: liquid–liquid phase separation
PrP^C^: cellular prion protein
PSCMA: poly(4-styrenesulfonic acid-*co*-maleic acid)
Aβo: amyloid-β oligomer
AD: Alzheimer’s disease
FRAP: fluorescence recovery after photobleaching
HSQC: heteronuclear single quantum coherence
LCD: low-complexity domain
OR: octarepeat
SEC: size-exclusion chromatography
