# Supplementary material for: Cellular Prion Protein Conformational Shift after Liquid–Liquid Phase Separation Regulated by a Polymeric Antagonist and Mutations
Source: J Am Chem Soc. 2024 Sep 26;146(40):27903–14. doi: 10.1021/jacs.4c10590 (PMC11469297; doi:10.1021/jacs.4c10590)
Supplement: Supplementary file 1 — ja4c10590_si_001.pdf [file ja4c10590_si_001.pdf]

Supporting Information

Cellular Prion Protein Conformational Shift after Liquid-Liquid Phase Separation Regulated by Polymeric Antagonist and Mutations

Yangyi Liu[1,2], Marcus D. Tuttle[1], Mikhail A. Kostylev[2], Graham P. Roseman[2], Kurt W. Zilm[1,*,#], and Stephen M. Strittmatter[2,*,#]

[1]Department of Chemistry, Yale University, 225 Prospect Street, New Haven, CT 06511, United States.
[2]Departments of Neuroscience and Neurology, Yale School of Medicine, 100 College Street, New Haven, CT 06510, United States.
[*]Corresponding authors. Emails: kurt.zilm@yale.edu; stephen.strittmatter@yale.edu
[#]K.W.Z. and S.M.S. contributed equally to this work.

Experimental Methods

Recombinant PrP^C expression and purification

Recombinant PrP^C was produced as described.[1,2] PrP plasmid in pRSET-A vector contains an N-terminal 6His-tag and a thrombin cleavage site. Mutagenesis was performed with Q5 Site-Directed Mutagenesis Kit (NEB). Overnight growth of BL21(DE3) in non-inducing medium MDAG135 was used as the starter culture, followed by a larger-volume overnight growth at room temperature in LB5052 autoinduction medium containing 1:1000 trace metals (Teknova, T1001), 2 mM $MgSO_4$, 100 µg/mL ampicillin, 1x salt M and 1x 5052 autoinduction sugar mix.[3]

For the production ^{15}N, ^{13}C-labeled PrP, overnight starter culture was centrifuged at 3000 x g for 7 min and resuspended in minimal media containing 1:1000 trace metals, 100 µg/mL ampicillin, 2 mM $MgSO_4$, 0.32% m/v $^{15}NH_4Cl$, 0.25% m/v ^{13}C-glucose, 50 mM Na_2HPO_4, and 50 mM KH_2PO_4. Culture was allowed to grow overnight at room temperature and was thereafter treated with the same protocol as natural abundance samples.

The culture was then centrifuged for 30 min at 4000 x g, and the pellet fraction was lysed in Buffer G (6 M Guanidine HCl, 10 mM reduced glutathione, 100 mM Na_2HPO_4, 10 mM Tris-HCl, pH 8). The lysate was centrifuged at 17700 x g for 90 min, and the resulting supernatant was loaded onto HisPur Ni-NTA resin (Qiagen) in a gravity flow chromatography column. The resin was washed with 4 resin volume of Buffer G, followed by a 20% to 100% stepwise gradient of Buffer B (100 mM Na_2HPO_4, 10 mM Tris-HCl, pH 8) in Buffer G for protein refolding. After a 50 mM imidazole wash in Buffer B, the protein was eluted with 600 mM imidazole in 10 mM Na_2HPO_4, pH 5.8. Eluted protein was concentrated using 3 kDa cutoff Amicon spin filters and dialyzed in 3.5 kDa Slide-A-Lyzer cassettes (ThermoFisher), in 5 mM Na_2HPO_4, pH 5.8 for 2 hours, and then overnight in water. The samples were treated overnight with thrombin at 1 Unit/mg to remove the 6His-tag. Cation exchange was performed chromatography using Source 15S column in AKTApure FPLC system. The column was equilibrated in 10 mM Na_2HPO_4, pH 6.5, and the protein was eluted in a 0-100% 20 CV gradient of 1M NaCl, 10 mM Na_2HPO_4, pH 6.5 at 10 mL/min. PrP^C was collected based on A280 in a single peak on the chromatogram and dialyzed in 3.5 kDa cassettes in 5 mM Na_2HPO_4, pH 5.8 for 2 hours, and then overnight in water.

Fluorescent PrP^C Labeling

Fluorescent PrP labeling was prepared using a protocol adapted from Kostylev et al., 2018.[1] 6His-tagged PrP of a concentration of 25 µM was mixed with 2x molar of Alexa Flour 488 NHS ester (Sigma-Aldrich 41698) in PBS overnight at room temperature. The resulting mixture was purified with HisPur Ni-NTA resin (Qiagen) in a gravity column. PBS washes were repeated 3-5 times until the flowthrough was free of unreacted dye. The labeled protein was eluted with 600 mM imidazole in 10 mM Na_2HPO_4, pH 5.8 and dialyzed overnight twice in water before use.

Microscope imaging

Brightfield images of LLPS liquid droplets were taken under Zeiss LSM 900 with 40x oil objective. 100 µL of 200 µM protein samples were loaded onto 8-well chambered glass (ThermoFisher, 155360). LLPS was induced by addition of 10x PBS to achieve 1x final or PSCMA stock solution to achieve 1:8 (PSCMA:PrP) concentration ratio. Fluorescent imaging of PrP-PSCMA titration was performed in a black 96-well plate (Thermo Scientific 165305) using ImageXpress Micro Confocal 10x objective.

Fluorescence Recovery After Photobleaching (FRAP)

FRAP experiments were performed on Perkin Elmer Ultraview VoX spinning disk confocal microscope with PhotoKinesis FRAP unit under 60x oil immersion objective. Regions of interest (ROIs) of 2x2 µm squares were photobleached on 120x120 µM imaging areas. Images were collected every second for 7

seconds before the photobleaching. Following the photobleaching, images were collected every second for the first 30 seconds and every 4 seconds for an additional 900 seconds or 7200 seconds, depending on the rate of recovery of each sample. Quantification of fluorescence intensity were performed in Velocity software (Perkin Elmer).

Solution NMR spectroscopy

Dialyzed ^{15}N, ^{13}C-PrP was concentrated with 3kD cutoff Amicon filters to achieve a concentration of 200 μM for optimal spectral resolution. NaOH and HCl were used for pH adjustments. PSCMA was dialyzed twice overnight in 3.5 kD cutoff cassettes in water, and its concentration was monitored by A262 measurements before adding to PrP. Each sample was supplemented with 10% v/v D_2O. ^{15}N-^{1}H TROSY HSQC and ^{13}C 1D spectra were taken at 18.8 T (Agilent 800 MHz NMR spectrometer, H{CN}^{13}C-sensitivity enhanced salt-tolerant cold probe) using BioPack pulse sequences (watergate-TROSY-^{15}N-^{1}H-HSQC and one-pulse-^{13}C). ^{13}C 1D spectra were processed with VNMRJ with baseline corrections. ^{15}N-^{1}H HSQC spectra were processed with NMRPipe and analyzed with NMRFAM-Sparky on the platform NMRBox.[4,5]

Solid-State NMR spectroscopy

Dialyzed ^{15}N, ^{13}C-PrP^{C} was concentrated with 3kD cutoff Amicon filters to achieve a concentration higher than 330 μM, followed by LLPS induction with the addition of either 10% v/v 10x PBS or dialyzed PSCMA to achieve a 1:8 concentration ratio (PSCMA:PrP^{C}). The liquid layer was packed into a 2.5 mm rotor by ultracentrifugation at 104,677 x g for 30 min using an in-house packing tool. All steps mentioned above were performed at 4 °C. Direct-polarized ^{13}C 1D spectrum of each sample was taken at 18.8 T (Agilent 800 MHz NMR Spectrometer, custom built triple resonance ^{1}H,^{13}C,^{15}N ssNMR probe[6]). Magic angle spinning was applied by an Agilent MAS controller to 17,700 Hz. Temperature was controlled with room temperature air and a Ranque-Hilsch vortex tube cooler.[7] Adamantane was used as an external reference.[8] Spectra were processed in VNMRJ.

PrP^{C} plate-based binding assay

This assay was adapted from a previously published protocol [1,9]. White 384-well MaxiSorp microplates (Thermofisher, 460372) were coated overnight with 20 μL/well 250 mM PrP^{C} in a coating buffer (30 mM Na_2CO_3, 80 mM $NaHCO_3$, pH 9.6). These plates were washed with 3 dispensing/aspiration cycles of 100 μL/well PBST (PBS and 0.05% Tween 20) using a BioTek 405 LS plate washer (Agilent) and incubated overnight with 20μL/well 2-fold serial dilutions of Alexa594-PSCMA (Akina Inc.) in PBSTB (PBST with 0.5% BSA) at 4 °C. A final PBST wash was performed before fluorescence reading was acquired with Victor Nivo plate reader (Perkin Elmer).

LLPS turbidity assay

Multiple mutants of PrP^{C} were tested for their abilities to phase separate in PSCMA. Samples of 200 μM PrP^{C} solution were prepared in 96-well plates (Corning, 353072), and LLPS was evaluated through a turbidity measurement (A595) with Victor 3V plate reader (Perkin Elmer). PSCMA solution of 250 μM was sequentially added to the protein solution to achieve different concentration ratios, and the A595 measurement was taken after each PSCMA addition. The kinetic rescue experiment was performed as the following: five wells of 200 μM PrP^{C} was induced by PSCMA at 1:8 (PSCMA:PrP^{C}) at time 0 and was incubated for a certain time at room temperature. After 1, 5, 20, 60, or 1080 minutes, the second dose of PSCMA was added to each sample respectively to achieve a 1:1 concentration ratio attempting to rescue the liquid back to solution, which was examine by measuring A595.

Size-exclusion chromatography

The composition of condensed and dilute phases of PSCMA-induced PrP^C LLPS was analyzed with SEC. Two 200 μM PrP^C solution samples were induced by PSCMA at 1:8 concentration ratio. The two samples were treated exactly the same except the temperature of an overnight incubation after induction. Following the incubation, each sample was centrifuged at 18,213 x g at 4 °C for 30 min, and the pellet was treated with a suspension buffer (8 M urea, 200 mM Tris) to reach the same volume as the supernatant fraction. Each fraction was loaded onto Superdex 75 in AKTApure FPLC system at a flow rate of 1 mL/min with 200 mM Tris, pH 7.4, monitored by A228, A280, and A575 readings. Fractions were collected at 1 mL throughout the entire run, and 100 μL of each fraction was loaded onto a 96-well plate to acquire a fluorescence reading with Victor 3V plate reader.

Supplementary Figures

```
                                         23                           50
WT              MHHHHHHVNSGLVPR GSKKRPKPGGWNTGGSRYPGQGSPGGNRYP
7K              MHHHHHHVNSGLVPR GSAARPAPGGWNTGGSRYPGQGSPGGNRYP
D167N,E168Q     MHHHHHHVNSGLVPR GSKKRPKPGGWNTGGSRYPGQGSPGGNRYP
E200K           MHHHHHHVNSGLVPR GSKKRPKPGGWNTGGSRYPGQGSPGGNRYP
E200Q           MHHHHHHVNSGLVPR GSKKRPKPGGWNTGGSRYPGQGSPGGNRYP
ΔOR             MHHHHHHVNSGLVPR GSKKRPKPGGWNTGGSRYPGQGSPGGNRYP

                51                                               100
WT              PQGGGGWGQPHGGGWGQPHGGGWGQPHGGGWGQPHGGGWGQGGGTHSQWN
7K              PQGGGGWGQPHGGGWGQPHGGGWGQPHGGGWGQPHGGGWGQGGGTHSQWN
D167N,E168Q     PQGGGGWGQPHGGGWGQPHGGGWGQPHGGGWGQPHGGGWGQGGGTHSQWN
E200K           PQGGGGWGQPHGGGWGQPHGGGWGQPHGGGWGQPHGGGWGQGGGTHSQWN
E200Q           PQGGGGWGQPHGGGWGQPHGGGWGQPHGGGWGQPHGGGWGQGGGTHSQWN
ΔOR             PQGGGGWGQ--------------------------------GGGTHSQWN

                101                                              150
WT              KPSKPKTNMKHMAGAAAAGAVVGGLGGYMLGSAMSRPIIHFGSDYEDRYY
7K              APSAPATNMAHMAGAAAAGAVVGGLGGYMLGSAMSRPIIHFGSDYEDRYY
D167N,E168Q     KPSKPKTNMKHMAGAAAAGAVVGGLGGYMLGSAMSRPIIHFGSDYEDRYY
E200K           KPSKPKTNMKHMAGAAAAGAVVGGLGGYMLGSAMSRPIIHFGSDYEDRYY
E200Q           KPSKPKTNMKHMAGAAAAGAVVGGLGGYMLGSAMSRPIIHFGSDYEDRYY
ΔOR             KPSKPKTNMKHMAGAAAAGAVVGGLGGYMLGSAMSRPIIHFGSDYEDRYY

                151                                              200
WT              RENMHRYPNQVYYRPMDEYSNQNNFVHDCVNITIKQHTVTTTTKGENFTE
7K              RENMHRYPNQVYYRPMDEYSNQNNFVHDCVNITIKQHTVTTTTKGENFTE
D167N,E168Q     RENMHRYPNQVYYRPMNQYSNQNNFVHDCVNITIKQHTVTTTTKGENFTE
E200K           RENMHRYPNQVYYRPMDEYSNQNNFVHDCVNITIKQHTVTTTTKGENFTK
E200Q           RENMHRYPNQVYYRPMDEYSNQNNFVHDCVNITIKQHTVTTTTKGENFTQ
ΔOR             RENMHRYPNQVYYRPMDEYSNQNNFVHDCVNITIKQHTVTTTTKGENFTE

                201                         230
WT              TDVKMMERVVEQMCITQYERESQAYYQRGS
7K              TDVKMMERVVEQMCITQYERESQAYYQRGS
D167N,E168Q     TDVKMMERVVEQMCITQYERESQAYYQRGS
E200K           TDVKMMERVVEQMCITQYERESQAYYQRGS
E200Q           TDVKMMERVVEQMCITQYERESQAYYQRGS
ΔOR             TDVKMMERVVEQMCITQYERESQAYYQRGS
```

Figure S1. Amino acid sequence of WT PrP and mutants used in this study. N-terminal peptide in grey was cleaved off by thrombin digestion in all preparations.

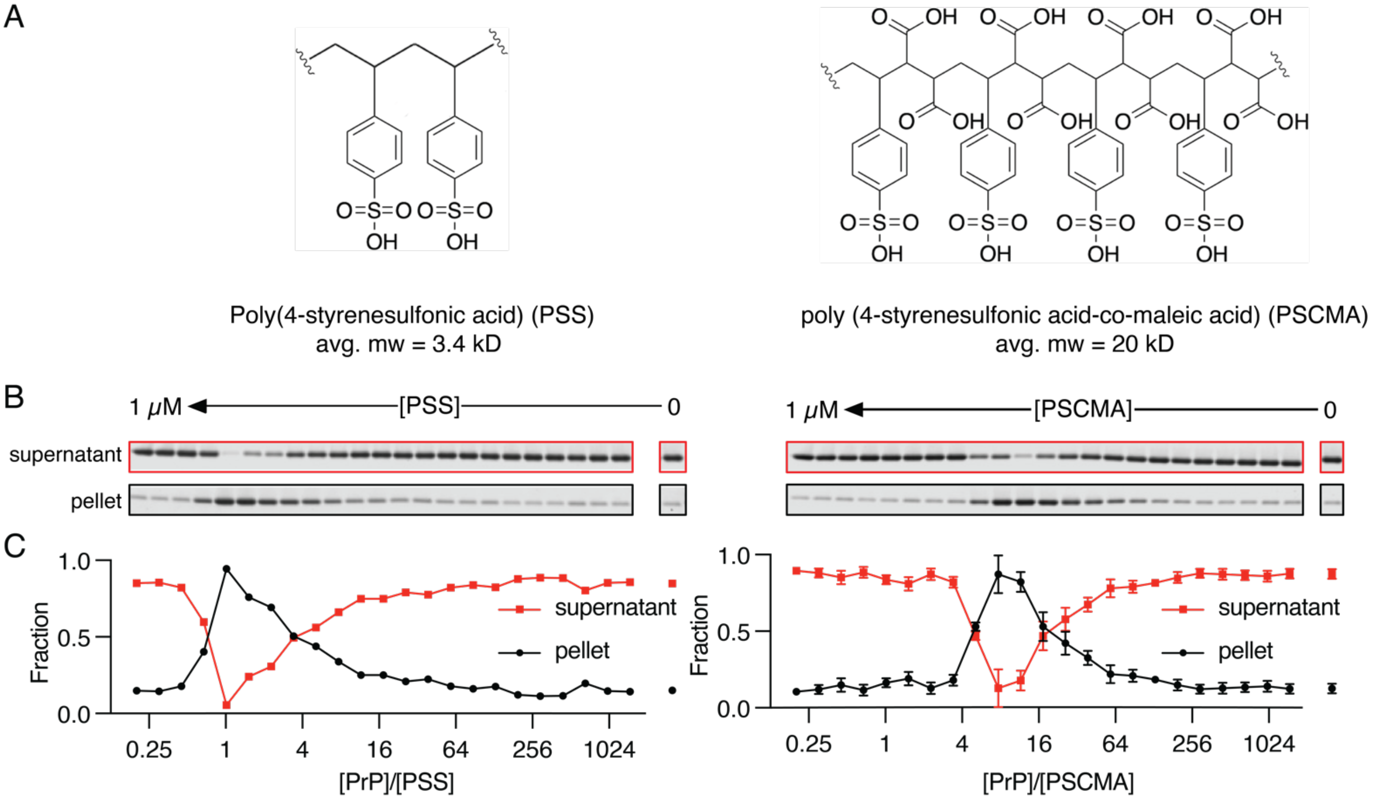

Figure S2. Lower molecular weight poly (4-styrenesulfonic acid) (PSS) induces reentrant LLPS of PrP^C but at concentration ratios different from PSCMA. (A) Structures of PSS (left) and PSCMA (right). (B) SDS-PAGE analysis of supernatant and pellet fractions of a PSS-PrP^C titration (left) in comparison to PSCMA-PrP^C (right). (C) Quantification of PrP^C band in supernatant (black) and in pellet fractions (red) in B; PSS (left) induces maximal LLPS of PrP^C at ~1:1 molar ratio, versus PSCMA (right) at ~1:8.

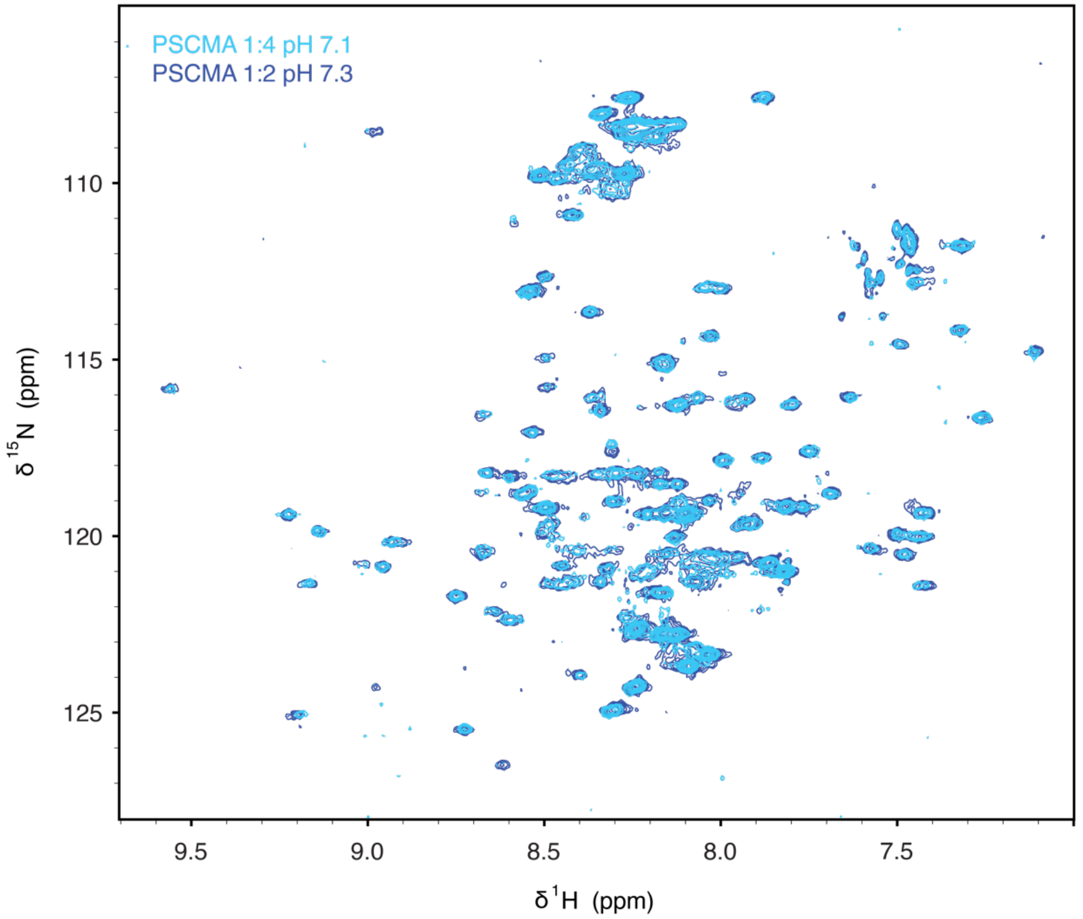

Figure S3. ^{15}N-^{1}H HSQC spectral overlay of 1:4 and 1:2 samples acquired at 800 MHz showing PrP^{C} has the same conformation in the two conditions beyond the critical 1:8 concentration ratio regardless of pH difference.

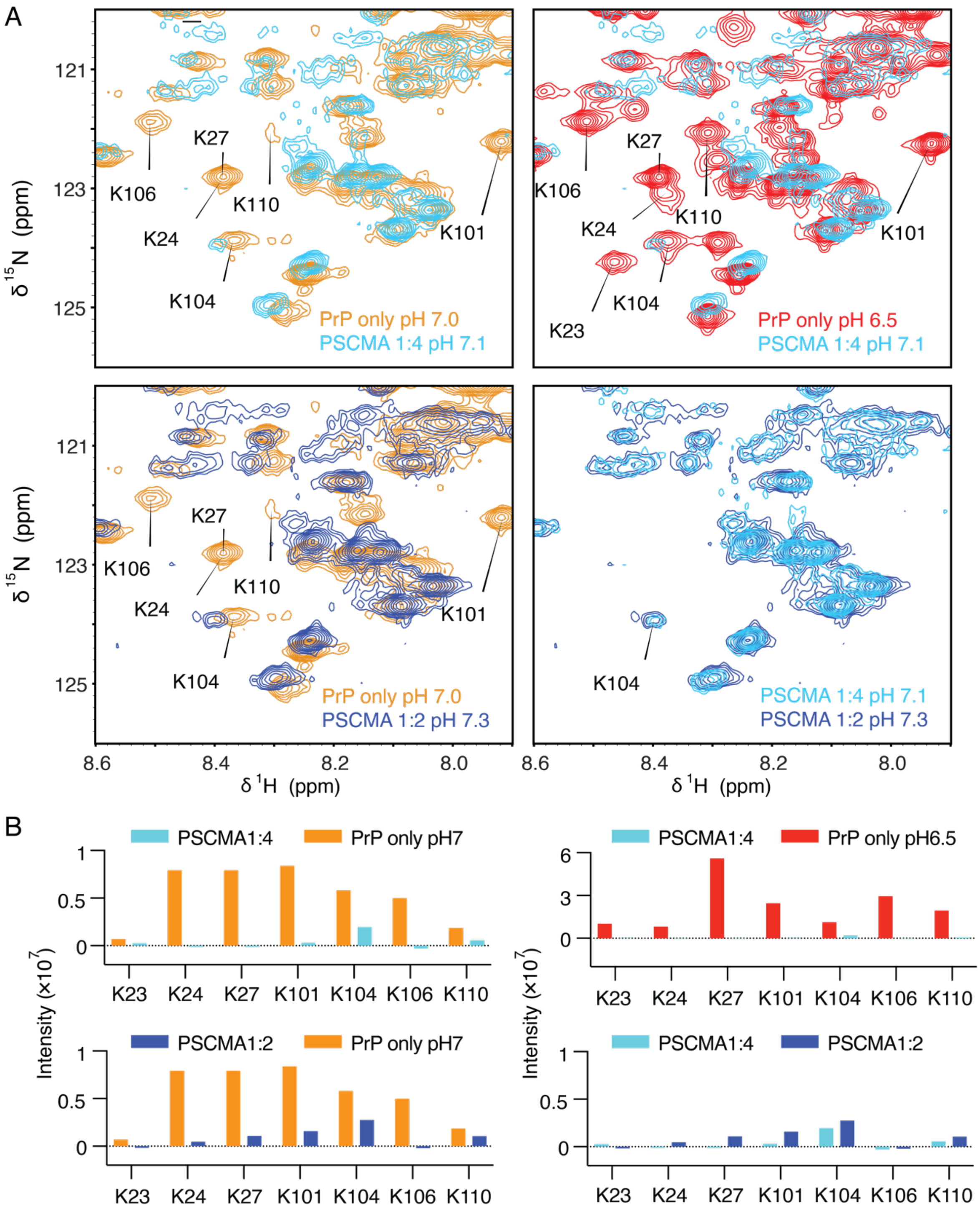

Figure S4. N-terminal lysine residues disappear due to interaction with PSCMA but not mere pH change. (A) Zoom in of ^{15}N-^{1}H HSQC pairwise spectral overlay of apo PrP^{C} and PSCMA-PrP^{C} complex at different pH values. Most lysine residues disappear only when PSCMA was present. (B) Comparison of peak intensity of each lysine residue under the conditions in (A).

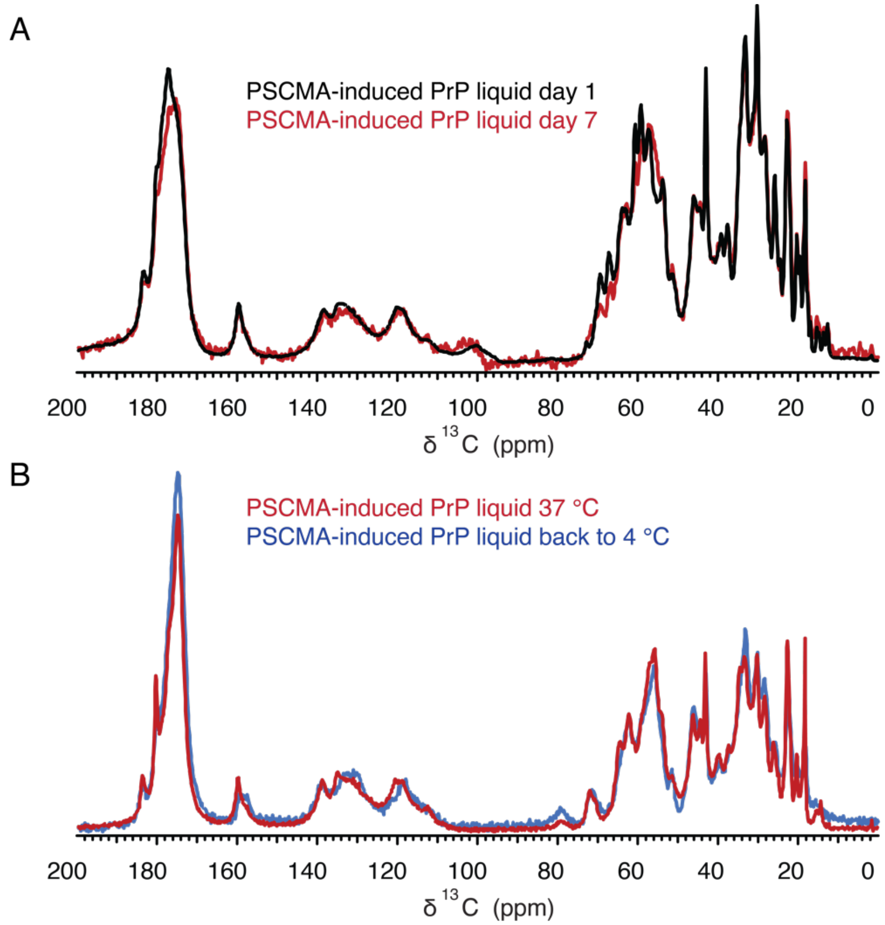

Figure S5. Maturation of PSCMA-induced PrP^C liquid requires heat and is irreverible. (A) PrP conformation remains relatively stable over 7 days at 4 °C. (B) Maturation happened when temperature increased and was irreversible by returning the temperature to 4 °C.

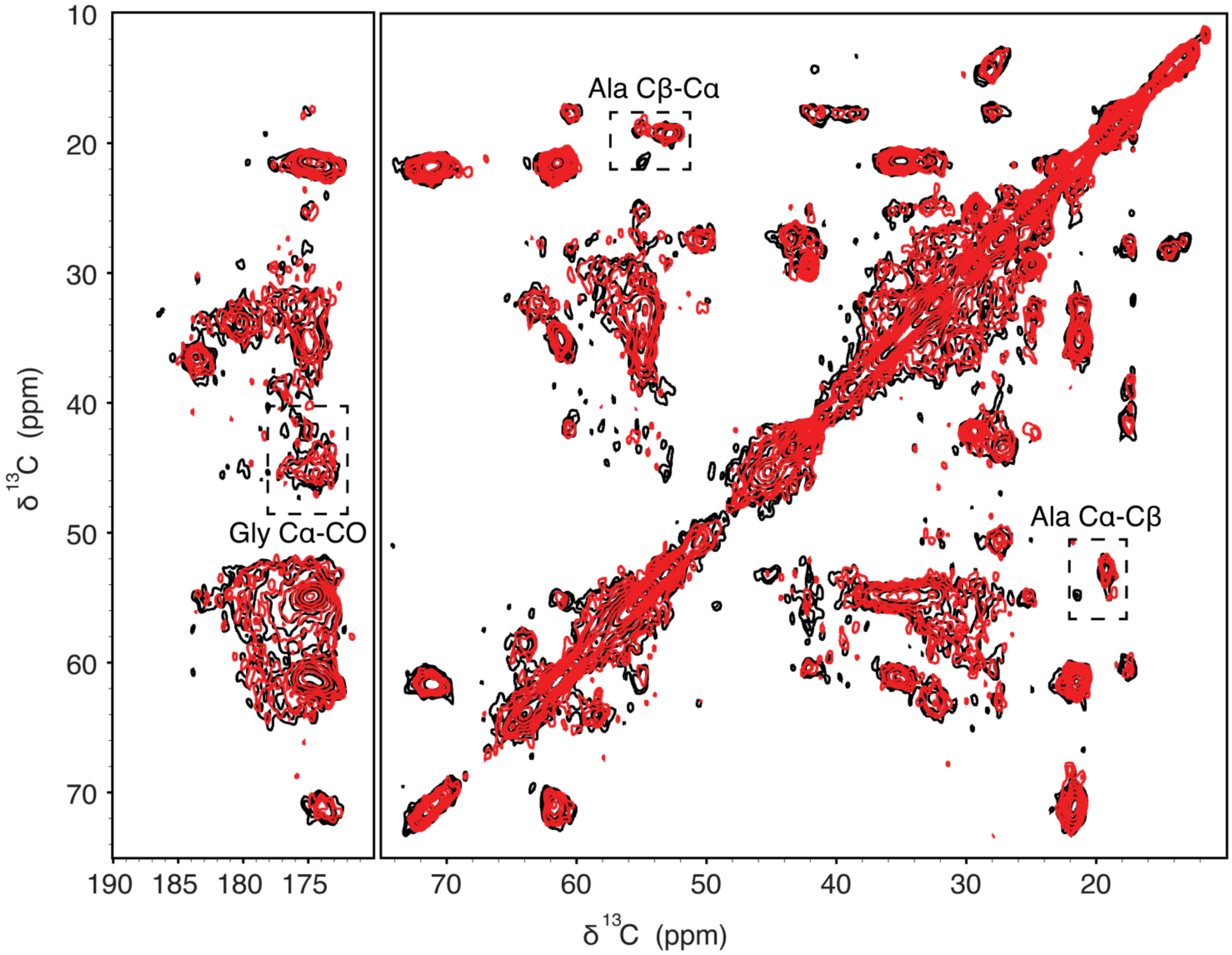

Figure S6. Overlay of ^{13}C-^{13}C 2D correlation ssNMR spectra of two matured liquids induced by either PBS (black) or PSCMA (red), further showing PrP has the same conformation in the two conditions.

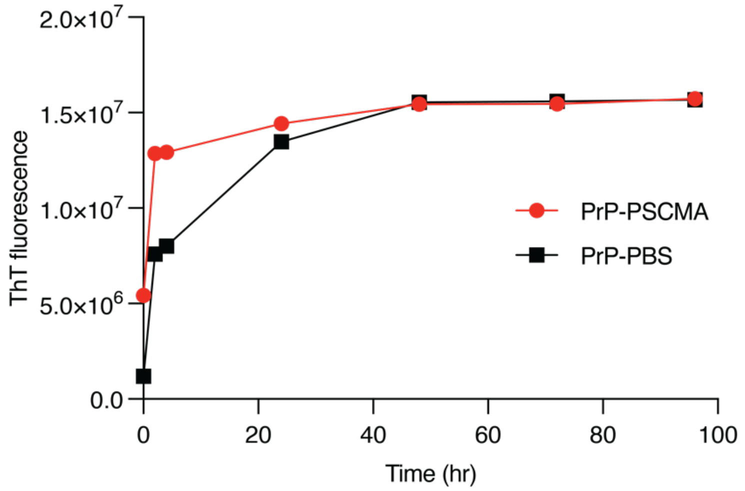

Figure S7. ThT fluorescence signal of both PSCMA- (red) PBS-induced (black) PrP^C liquid increases upon 37 °C incubation. PrP solution was supplemented with ThT prior to induction. For induction, PSCMA was added to achieve 1:8 concentration ratio, and 10x PBS was added to achieve 1x PBS in the final mixture. Final concentration of PrP is identical at 98 μM and ThT at 20 μM. The two liquids eventually have the same signal intensity, suggesting that they have similar conformations after maturation, consistent to ssNMR results.

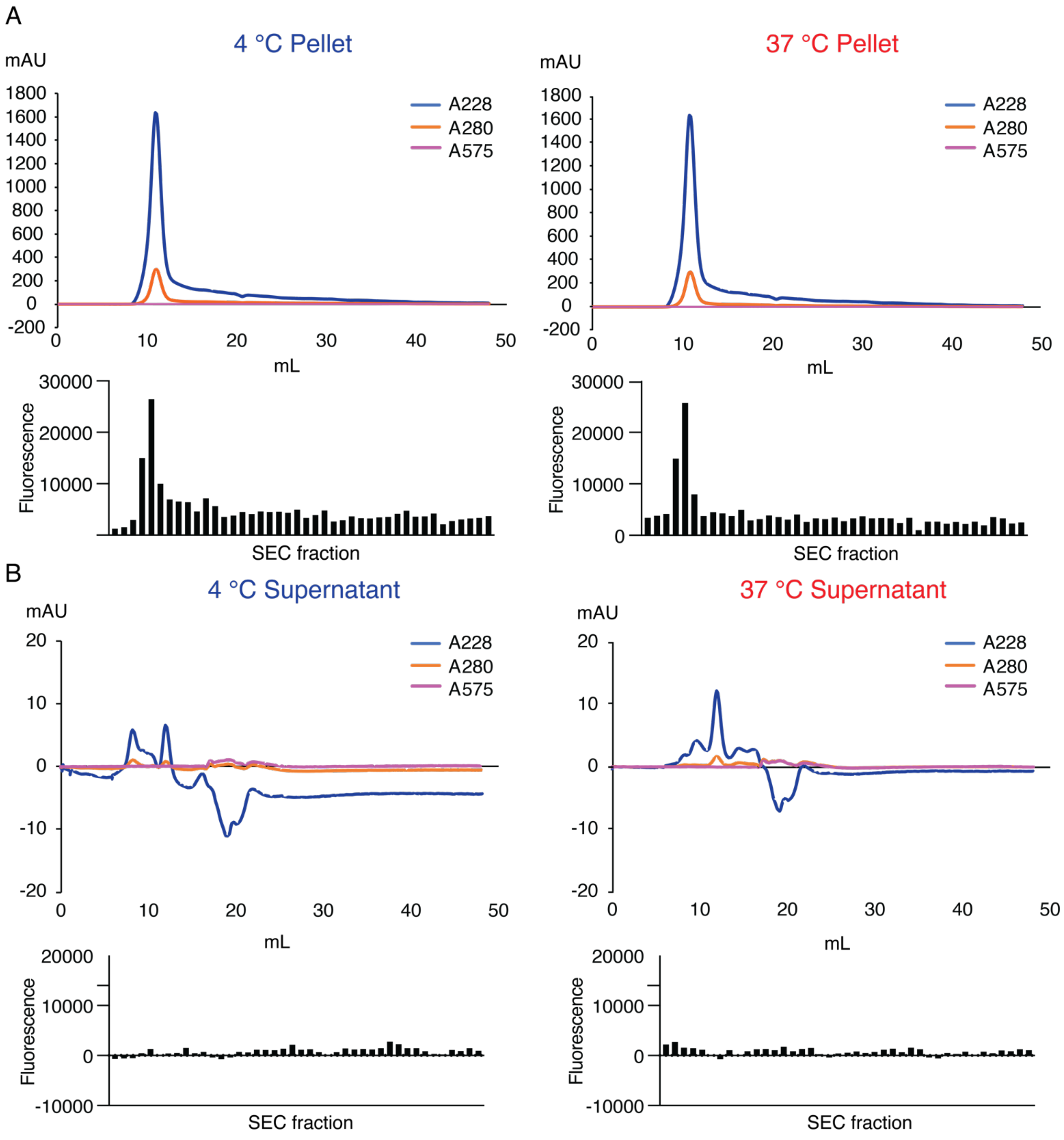

Figure S8. Size exclusion chromatography (SEC) analysis on fluorescently labeled PSCMA-induced PrP^C liquid at 1:8 ratio that has undergone either 4 or 37 °C incubation showing maturation does not exclude PSCMA from the liquid phase. (A) Analysis of the pellet fraction of the two samples. Chromatogram (top) and the fluorescence signal (bottom) of each 1 mL fraction showing both the protein and PSCMA stay in the condensed liquid phase regardless of maturation. (B) Similar analysis on the supernatant fraction shows no significant protein absorbance or PSCMA fluorescence signal.

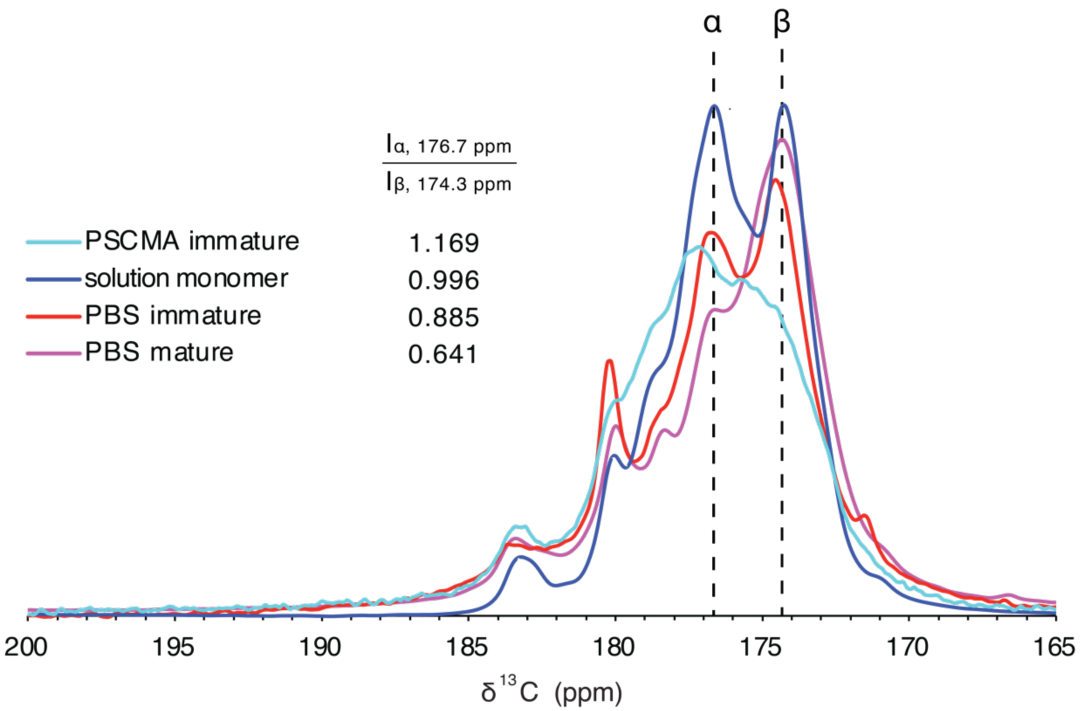

Figure S9. Overlay of ^{13}C ssNMR spectra of mature condense liquid phase (magenta), initial condense liquid phase (red), and PSCMA-induced initial condense liquid phase (cyan) and solution NMR spectrum of PrP^{C} monomeric state with exponential line broadening (150 Hz, blue). Intensity ratio of each spectrum at 176.7 ppm to 174.3 ppm was shown to indicate the ratio of amino acid residues in alpha-helical torsion angles relative to beta-sheet.

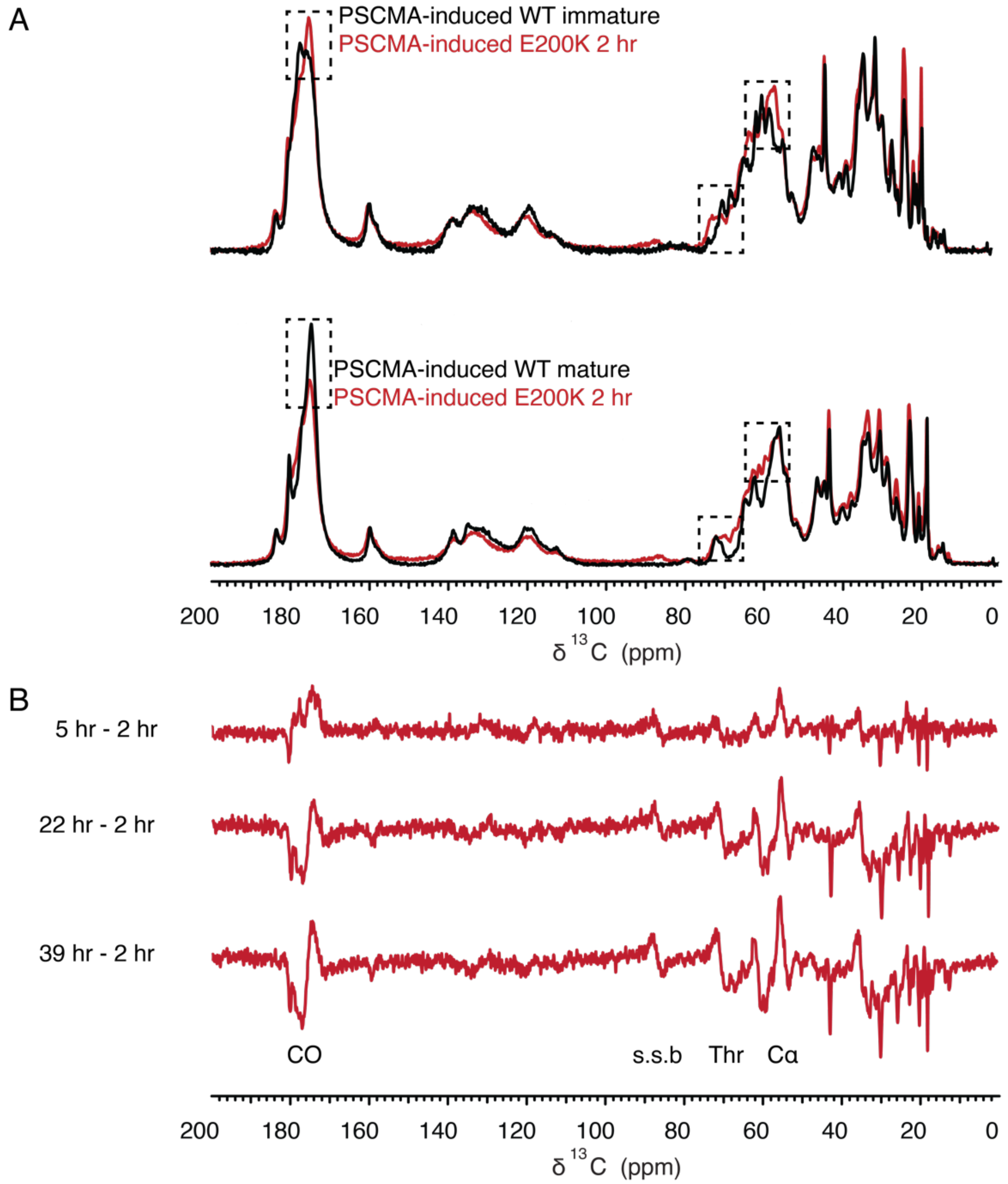

Figure S10. Maturation of PSCMA-induced E200K liquid is fast and leads to conformational changes similar to that of WT. (A) Overlay of the initial spectrum of PSCMA-induced E200K liquid at 2 hours (red) to PSCMA-induced WT liquid in its immature (black, top) and mature state (black, bottom), showing that the initial spectrum of E200K is more similar to the mature state of WT. (B) 3x spectral difference of PSCMA-induced E200K at different time points minus the initial spectrum, highlighting the changes in CO, Thr, and Cα regions.

References

(1) Kostylev, M. A.; Tuttle, M. D.; Lee, S.; Klein, L. E.; Takahashi, H.; Cox, T. O.; Gunther, E. C.; Zilm, K. W.; Strittmatter, S. M. Liquid and Hydrogel Phases of PrPC Linked to Conformation Shifts and Triggered by Alzheimer's Amyloid-β Oligomers. *Mol Cell* 2018, *72* (3), 426-443.e12. https://doi.org/10.1016/J.MOLCEL.2018.10.009.

(2) Zahn, R.; von Schroetter, C.; Wüthrich, K. Human Prion Proteins Expressed in Escherichia Coli and Purified by High-Affinity Column Refolding. *FEBS Lett* 1997, *417* (3), 400–404. https://doi.org/10.1016/S0014-5793(97)01330-6.

(3) Studier, F. W. Protein Production by Auto-Induction in High Density Shaking Cultures. *Protein Expr Purif* 2005, *41* (1), 207–234. https://doi.org/10.1016/j.pep.2005.01.016.

(4) Lee, W.; Tonelli, M.; Markley, J. L. NMRFAM-SPARKY: Enhanced Software for Biomolecular NMR Spectroscopy. *Bioinformatics* 2015, *31* (8), 1325–1327. https://doi.org/10.1093/bioinformatics/btu830.

(5) Maciejewski, M. W.; Schuyler, A. D.; Gryk, M. R.; Moraru, I. I.; Romero, P. R.; Ulrich, E. L.; Eghbalnia, H. R.; Livny, M.; Delaglio, F.; Hoch, J. C. NMRbox: A Resource for Biomolecular NMR Computation. *Biophys J* 2017, *112* (8), 1529–1534. https://doi.org/10.1016/j.bpj.2017.03.011.

(6) Martin, R. W.; Paulson, E. K.; Zilm, K. W. Design of a Triple Resonance Magic Angle Sample Spinning Probe for High Field Solid State Nuclear Magnetic Resonance. *Review of Scientific Instruments* 2003, *74* (6), 3045–3061. https://doi.org/10.1063/1.1571951.

(7) Martin, R. W.; Zilm, K. W. Variable Temperature System Using Vortex Tube Cooling and Fiber Optic Temperature Measurement for Low Temperature Magic Angle Spinning NMR. *Journal of Magnetic Resonance* 2004, *168* (2), 202–209. https://doi.org/10.1016/J.JMR.2004.03.002.

(8) Morcombe, C. R.; Zilm, K. W. Chemical Shift Referencing in MAS Solid State NMR. *Journal of Magnetic Resonance* 2003, *162* (2), 479–486. https://doi.org/10.1016/S1090-7807(03)00082-X.

(9) Kostylev, M. A.; Kaufman, A. C.; Nygaard, H. B.; Patel, P.; Haas, L. T.; Gunther, E. C.; Vortmeyer, A.; Strittmatter, S. M. Prion-Protein-Interacting Amyloid-β Oligomers of High Molecular Weight Are Tightly Correlated with Memory Impairment in Multiple Alzheimer Mouse Models. *Journal of Biological Chemistry* 2015, *290* (28), 17415–17438. https://doi.org/10.1074/JBC.M115.643577.
